# Supplementary material for: From Biomedical Datasets to Fairness-Aware Recommendations: An Integrated Data Orchestration Pipeline for Binary Clinical Predictions
Source: Comput Struct Biotechnol J. 2026 Sep 3;35(2):0215. doi: 10.34133/csbj.0215 (PMC13538956; doi:10.34133/csbj.0215)
Supplement: Supplementary file 1 [file csbj.0215.f1.zip › SI_1708.pdf]

Complete supplementary material for: From biomedical datasets to fairness-aware recommendations

Marta Alberola[a], Pedro Copado[a], Alfredo Vellido[a], Caroline König[a]

[a]*Intelligent Data Science and Artificial Intelligence (IDEAI-UPC) Research Centre, Universitat Politècnica de Catalunya - BarcelonaTech (UPC), Barcelona, Spain*

Purpose and scope

This supplementary material accompanies the revised manuscript and provides the supporting evidence package for the two case studies. It contains endpoint and cohort definitions, split summaries, subgroup-support tables, metric definitions, selected baseline results, mitigation summaries, statistical-evidence excerpts and graphical outputs generated during the dataset-specific executions. The ACTG175 case study is documented as one-year mortality prediction, with death by day 365 as the positive class; `cid` was not used as the endpoint variable. The stroke-risk case study is documented as a binary prediction task with `stroke`=1 as the positive class.

The supplementary package provides three layers of material. First, the PDF gives a readable curated summary of the analyses. Second, the machine-readable folders include extracted ACTG175 notebook tables, stroke-risk execution tables, source code, execution figures and a SHA-256 manifest for package contents. Third, selected execution figures are reproduced in the PDF so that the article is accompanied not only by numerical tables, but also by representative graphical outputs from the executed workflows.

1. Numerical summary aligned with the main manuscript

The values below reproduce the main numerical anchors used in the manuscript text, Table 1, Table 5, Table 6 and the corresponding manuscript figures. The ACTG175 values correspond to the executed death-only one-year mortality notebook. The stroke-risk values correspond to the exported stroke-risk execution artefacts.

Table 1: Baseline-performance values concordant with the main manuscript. BA = balanced accuracy; AP = average precision; ECE = expected calibration error.

Dataset	Selected model	Test N/events	Threshold	Accuracy	BA	ROC-AUC	AP	Brier	ECE	Recall	Precision	F1
ACTG175	Logistic regression	412/25	0.070	0.646	0.624	0.710	0.320	0.054	0.014	0.600	0.099	0.170
Stroke-risk	XGBoost	1,022/50	0.060	0.731	0.773	0.849	0.282	0.041	0.009	0.820	0.134	0.230

Table 2: Fairness and mitigation values concordant with the main manuscript.

Dataset	Item	Concordant value used in the manuscript
ACTG175	Endpoint	One-year mortality prediction; death by day 365 is the positive class; `cid` was not used as endpoint variable.
ACTG175	Cohort	2,058 analytic records; 123 one-year deaths; 5.98% event rate; 81 records excluded because one-year mortality status was not observable.
ACTG175	Fairness priority	`gender_label`; FPR gap 0.040, FNR gap 0.417, combined FPR+FNR gap 0.457.
ACTG175	Groupwise rates	Female FNR 0.750 and male FNR 0.333 at the selected baseline threshold.
ACTG175	Leading mitigation signal	ElasticNetLogistic + ThresholdOptimizer-DP; residual targeted gap 0.151; gap gain 0.190, corresponding to approximately 55.8% relative reduction; BA change +0.046 versus the logistic benchmark.
Stroke-risk	Cohort	5,110 analytic records; 249 stroke events; 4.87% event rate.
Stroke-risk	Single-attribute gaps	Age-group combined FPR+FNR gap 1.522; work-type combined FPR+FNR gap 1.521.
Stroke-risk	Leading mitigation signal	Random forest with Fairlearn demographic-parity threshold post-processing; aggregate combined FPR+FNR gap reduction 39.41%; mean BA change -0.002; aggregate bootstrap p = 0.00399.

2. Graphical outputs from the executed pipelines

This section provides selected graphical outputs from the executed ACTG175 and stroke-risk workflows. Together with the supplementary tables, these figures document cohort structure, model comparison, subgroup-disparity patterns, mitigation ranking and decision-support outputs for the two datasets.

2.1. ACTG175 one-year mortality execution

The ACTG175 figures reproduce selected outputs from the executed one-year mortality notebook. They cover subgroup support and split profile, model-family comparison, baseline subgroup-error patterns, mitigation screening and the final global top-6 comparison.

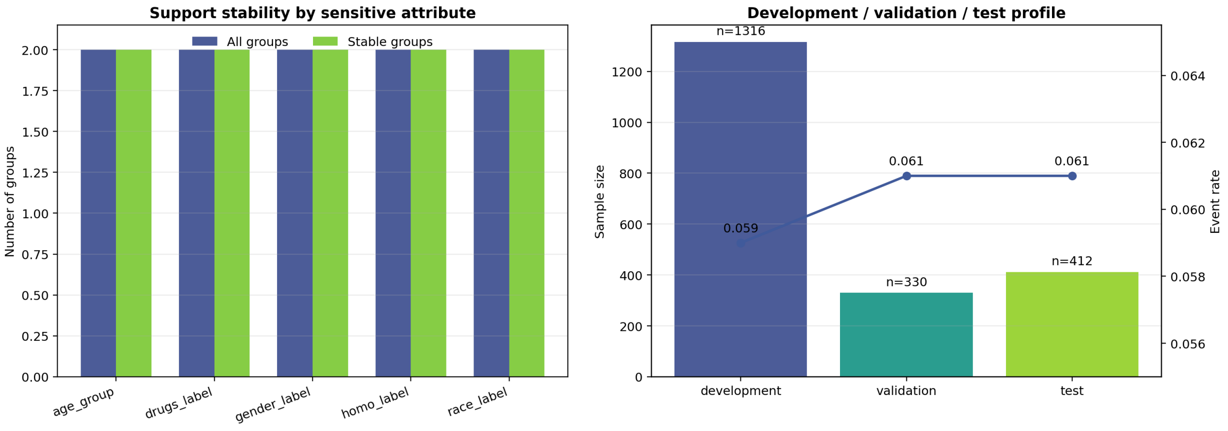

Figure 1: ACTG175 execution output showing support stability across sensitive attributes and the development, validation and test profile after endpoint construction.

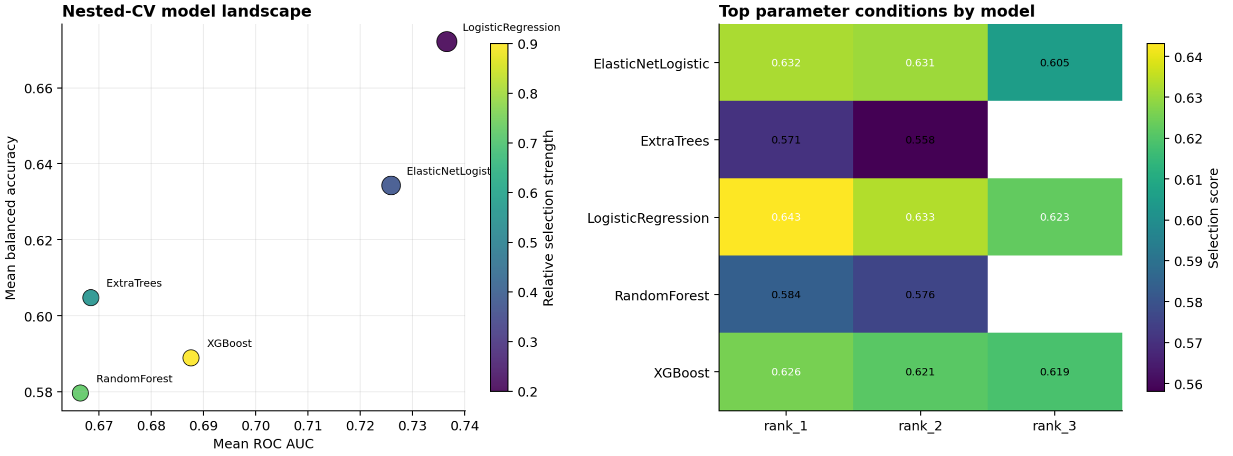

Figure 2: ACTG175 execution output summarising the nested cross-validation model landscape and the top parameter conditions by model family.

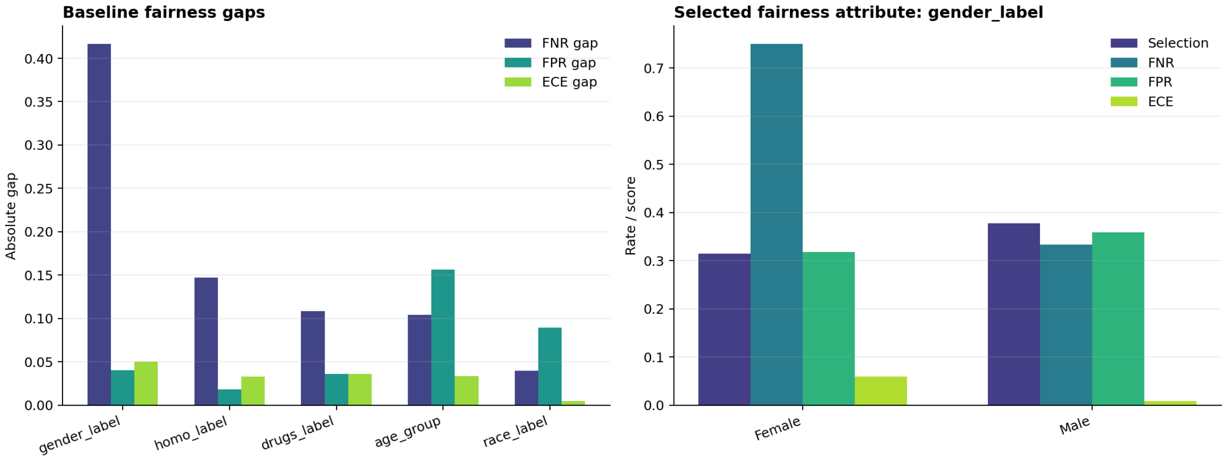

Figure 3: ACTG175 execution output showing baseline subgroup-error gaps and groupwise error rates for the selected fairness attribute at the chosen operating threshold.

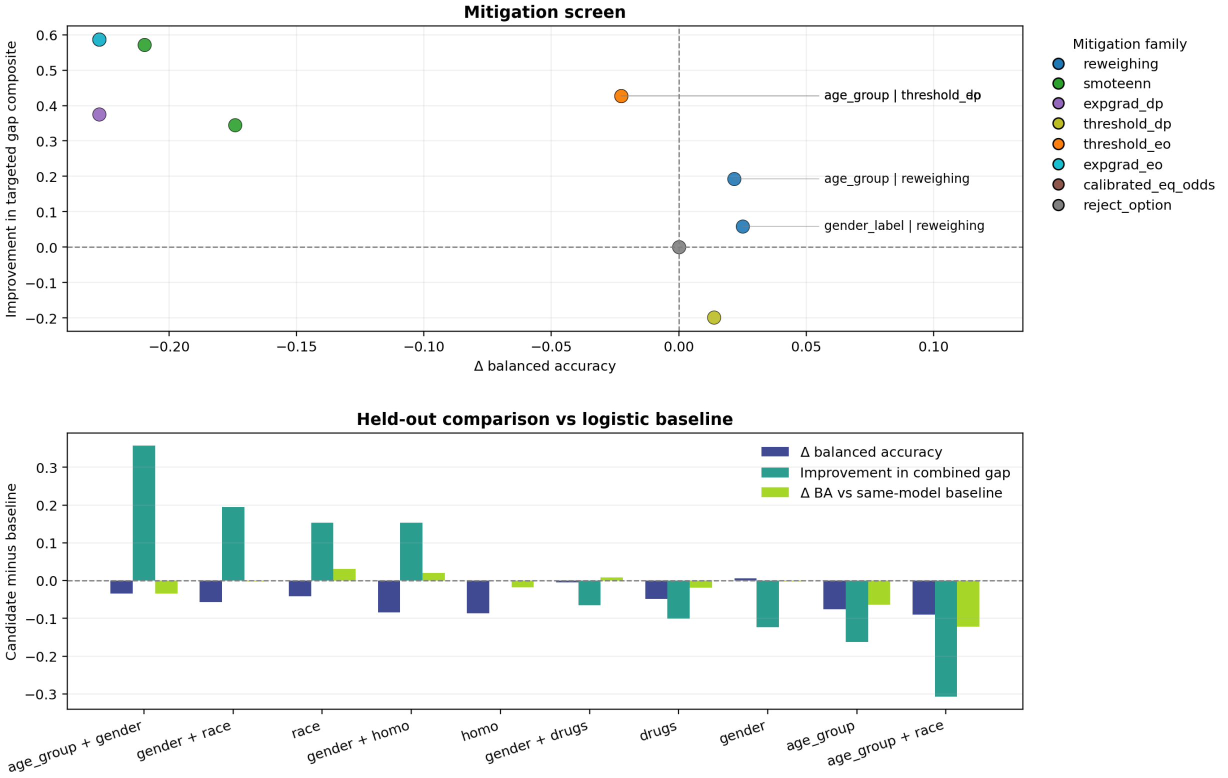

Figure 4: ACTG175 execution output showing mitigation screening and held-out comparison against the selected baseline.

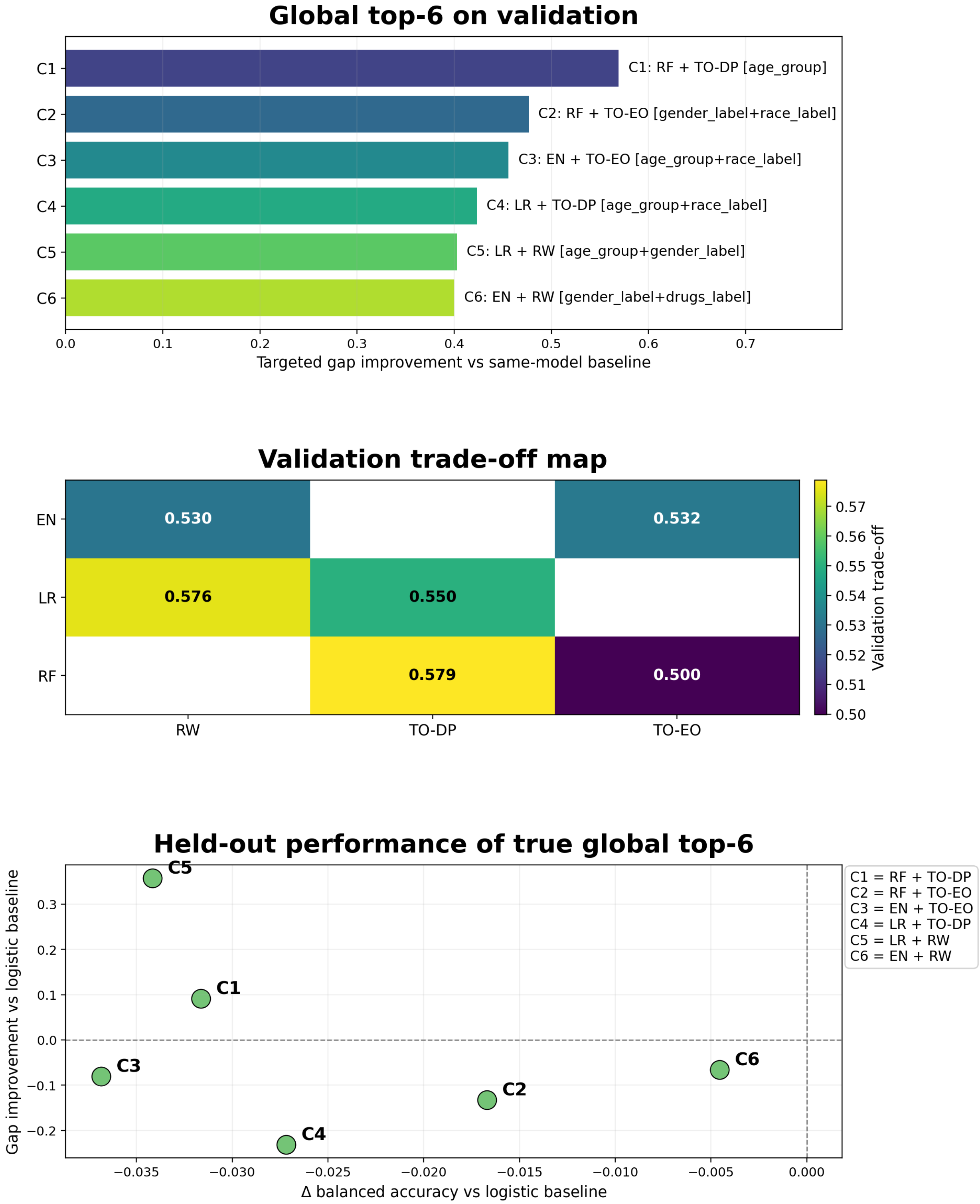

Figure 5: ACTG175 execution output summarising the final validation shortlist, validation trade-off table and held-out performance of the global top-6 candidates. Candidate codes are: C1 = RF + TO-DP, C2 = RF + TO-EO, C3 = EN + TO-EO, C4 = LR + TO-DP, C5 = LR + RW, and C6 = EN + RW; RF = RandomForest, EN = ElasticNetLogistic, LR = LogisticRegression, TO-DP = ThresholdOptimizer-DP, TO-EO = ThresholdOptimizer-EO, and RW = Reweighing.

2.2. Stroke-risk execution

The stroke-risk figures reproduce selected outputs from the executed stroke-risk workflow. They cover observed event rates across fairness-audit attributes, the largest subgroup gaps for the selected baseline, ROC behaviour across model families and the ranking of mitigation combinations.

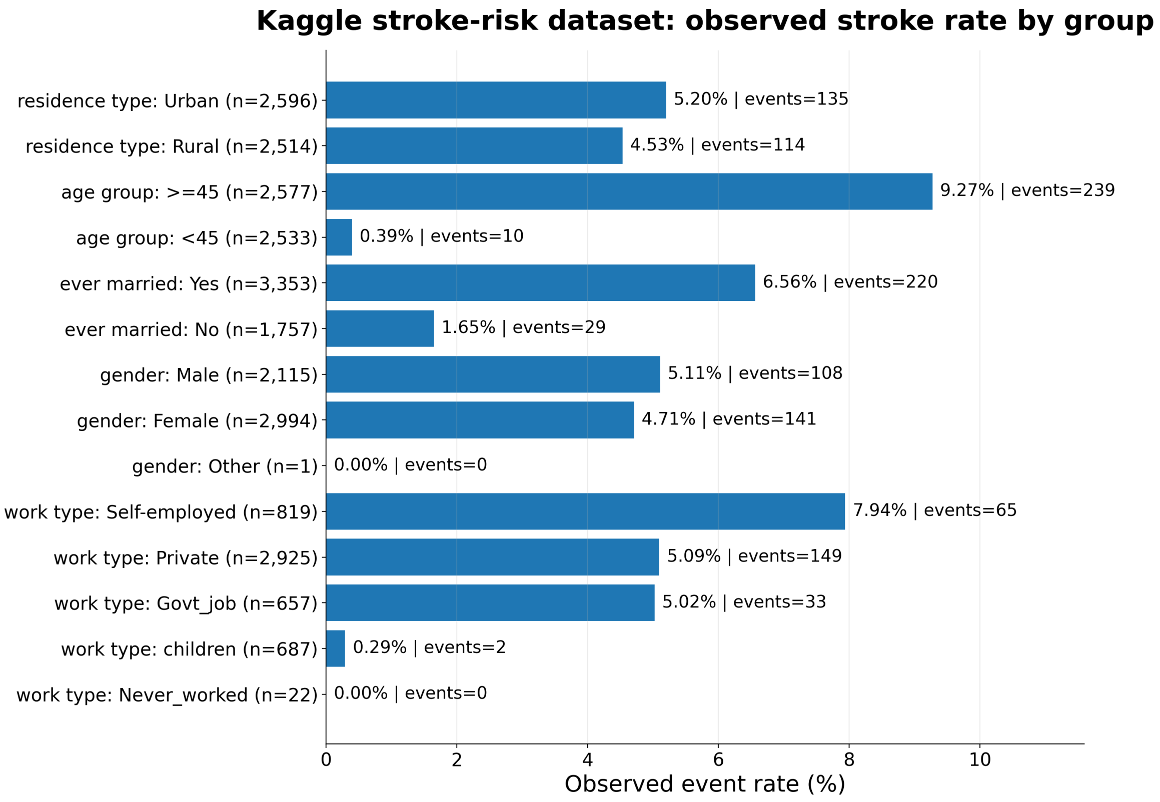

Figure 6: Stroke-risk execution output showing observed stroke event rates by sensitive or fairness-audit group.

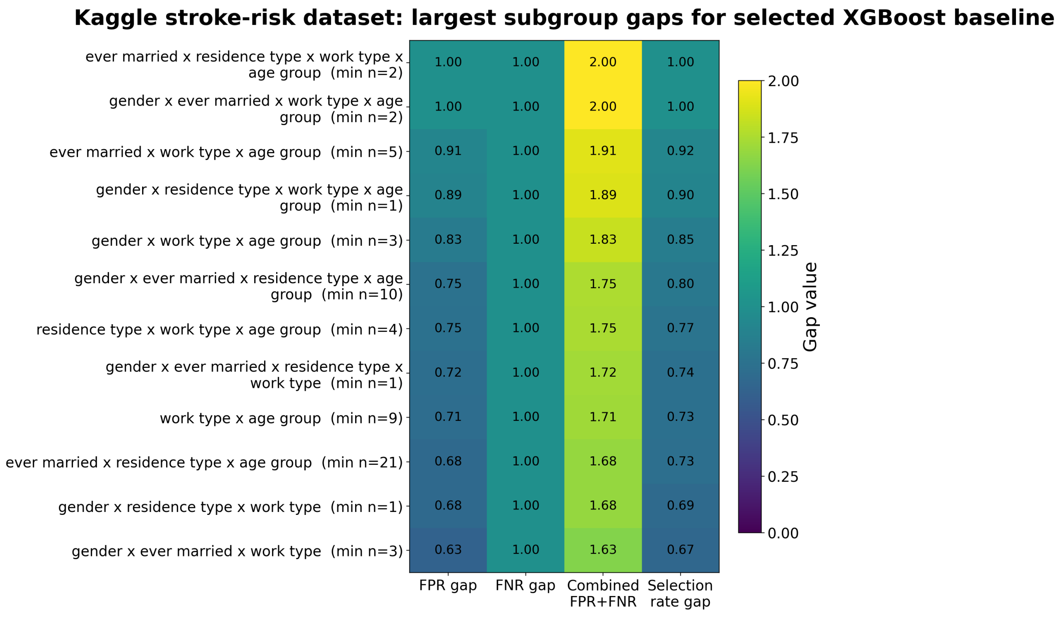

Figure 7: Stroke-risk execution output showing the largest subgroup gaps for the selected XGBoost baseline.

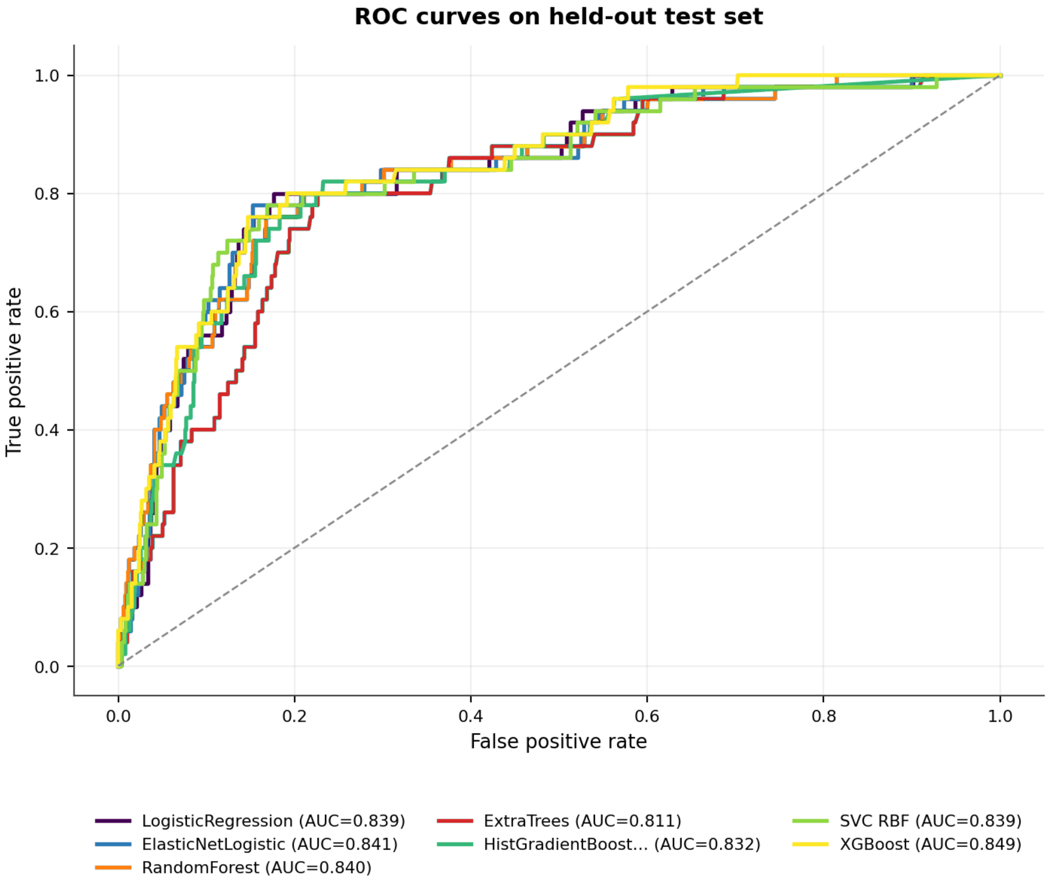

Figure 8: Stroke-risk execution output showing held-out ROC curves for the evaluated model families.

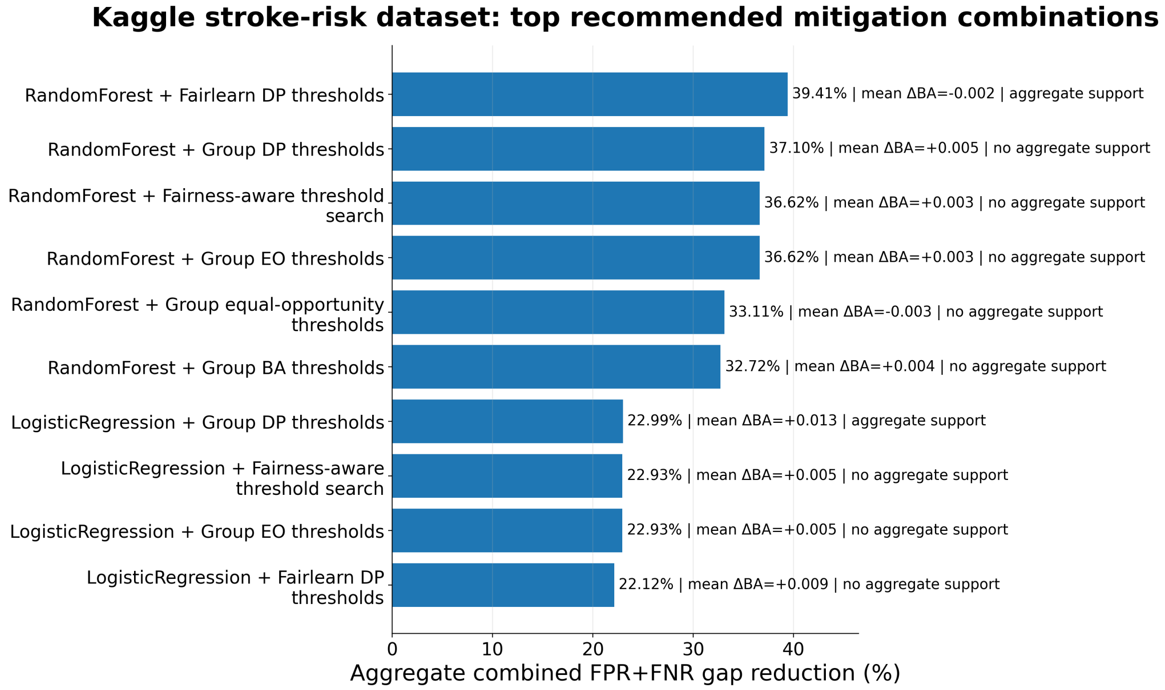

Figure 9: Stroke-risk execution output ranking the top recommended mitigation combinations by aggregate combined FPR+FNR gap reduction.

3. Supplementary package inventory

Table 3: Supplementary files provided with this package.

Component	Content
PDF/LaTeX supplement	Curated supplementary text, readable tables and selected execution figures.
ACTG175 tables	26 CSV tables extracted from the executed one-year mortality notebook.
Stroke tables	Numbered CSV artefacts from the stroke-risk execution.
Source code	Executed ACTG175 notebook, stroke-risk modular notebook and modular pipeline bundle.
Execution figures	Selected graphical outputs generated during the ACTG175 mortality notebook and stroke-risk pipeline executions.
Integrity manifest	SHA-256 digest and file size for the supplementary package contents.

4. Dataset provenance, endpoints and cohort flow

Raw-file hashes are treated as provenance evidence and are retained in the governed reproducibility package or repository manifest when available. The article and this supplement report source, access and endpoint information in a reader-facing format.

Table 4: Dataset provenance and endpoint definitions used in the article.

Dataset	Source/provenance	Endpoint	Analytic cohort	Note
ACTG175	UCI ACTG175 clinical-trial dataset; executed one-year mortality notebook.	Death by day 365; cid not used.	2,058 records; 123 events; 25 test events.	Source-copy hash evidence is retained in the governed local package or repository manifest when available.
Stroke-risk	Kaggle upload ranaghulamnabi/stroke-risk-dataset; accessed 5 June 2026.	stroke = 1.	5,110 records; 249 events; 50 test events.	No immutable Kaggle version ID or formal licence field was exposed in the available metadata.

Table 5: Cohort and target-flow summary.

Dataset	Raw rows	Excluded	Analytic rows	Events	Event rate
ACTG175	2,139	81 without observable one-year mortality status	2,058	123	5.98%
Stroke-risk	5,110	0 missing target rows	5,110	249	4.87%

Table 6: Train-validation-test split summary.

Dataset	Split	N	Events	Non-events	Event rate
ACTG175	train	1316	78	1238	0.059
ACTG175	validation	330	20	310	0.061
ACTG175	train_full	1646	98	1548	0.060
ACTG175	test	412	25	387	0.061
Stroke-risk	train	3270	159	3111	0.049
Stroke-risk	validation	818	40	778	0.049
Stroke-risk	train_full	4088	199	3889	0.049
Stroke-risk	test	1022	50	972	0.049

5. Subgroup support and fairness-audit attributes

Subgroup support was inspected before interpreting fairness gaps. Small subgroups and zero-event strata can make subgroup error estimates unstable.

Table 7: Sensitive-attribute support and event rates.

Dataset	Attribute	Group	N	Events	Event rate (%)
ACTG175	age_group	40+	567	47	8.290
ACTG175	age_group	<40	1491	76	5.100
ACTG175	drugs_label	No	1793	109	6.080
ACTG175	drugs_label	Yes	265	14	5.280
ACTG175	gender_label	Female	350	19	5.430
ACTG175	gender_label	Male	1708	104	6.090
ACTG175	homo_label	No	684	36	5.260
ACTG175	homo_label	Yes	1374	87	6.330
ACTG175	race_label	Non-white	578	32	5.540
ACTG175	race_label	White	1480	91	6.150
Stroke-risk	Residence_type	Rural	2514	114	4.535
Stroke-risk	Residence_type	Urban	2596	135	5.200
Stroke-risk	age_group	<45	2533	10	0.395
Stroke-risk	age_group	>=45	2577	239	9.274
Stroke-risk	ever_married	No	1757	29	1.651
Stroke-risk	ever_married	Yes	3353	220	6.561
Stroke-risk	gender	Female	2994	141	4.709
Stroke-risk	gender	Male	2115	108	5.106
Stroke-risk	gender	Other	1	0	0
Stroke-risk	work_type	Govt_job	657	33	5.023
Stroke-risk	work_type	Never_worked	22	0	0
Stroke-risk	work_type	Private	2925	149	5.094
Stroke-risk	work_type	Self-employed	819	65	7.937
Stroke-risk	work_type	children	687	2	0.291

6. Evaluation metrics

Table 8: Performance and fairness metrics used across the supplementary analyses.

Metric	Formula	Interpretation
Accuracy	(TP+TN)/N	Overall correct thresholded classifications; secondary in rare-event tasks.
Balanced accuracy	(TPR+TNR)/2	Primary thresholded utility metric balancing event and non-event recognition.
Recall / sensitivity	TP/(TP+FN)	Proportion of true events detected at the selected threshold.
FNR	FN/(TP+FN)	Missed-event proportion; audited by subgroup.
FPR	FP/(FP+TN)	False-alert proportion; audited by subgroup.
Precision / PPV	TP/(TP+FP)	Proportion of flagged records that are true events.
F1-score	2*P*R/(P+R)	Positive-class summary after threshold selection.
ROC-AUC	AUC(ROC)	Threshold-independent ranking performance.
Average precision	AUC(PR)	Positive-class-focused ranking performance.
Combined gap	FPRgap + FNRgap	Composite subgroup error-burden metric.

7. ACTG175 one-year mortality notebook outputs

These tables were extracted from the executed ACTG175 notebook used for the endpoint-corrected revision. The notebook selected logistic regression, isotonic probability calibration and a 0.07 operating threshold.

Table 9: ACTG175 subgroup support-threshold options.

min support	min events	stable groups	stable intersections	score	recommended	current
15	3	1	0.975	0.917	yes	no
20	5	1	0.900	0.860	no	no
30	10	1	0.800	0.750	no	yes

Table 10: ACTG175 model-family cross-validation summary.

model	rank	mean ROC	mean BA	Brier	selected
LogisticRegression	1	0.737	0.672	0.053	yes
ElasticNetLogistic	2	0.726	0.634	0.052	no
ExtraTrees	3	0.668	0.605	0.162	no
RandomForest	4	0.666	0.580	0.071	no
XGBoost	5	0.688	0.589	0.052	no

Table 11: ACTG175 probability-version and threshold-selection summary.

probability	threshold	BA	ROC	Brier	ECE	selected
isotonic	0.070	0.726	0.791	0.050	0.011	yes
calibrated_sigmoid	0.070	0.714	0.800	0.051	0.014	no
uncalibrated	0.080	0.713	0.800	0.051	0.023	no

Table 12: ACTG175 selected-baseline held-out metrics with bootstrap intervals.

metric	estimate	CI low	CI high	n boot
balanced_accuracy	0.624	0.552	0.651	8
roc_auc	0.710	0.608	0.787	8
brier	0.054	0.044	0.056	8
ece	0.014	0.016	0.024	8
fpr	0.351	0.305	0.401	8
fnr	0.400	0.306	0.538	8

The supplied execution used 8 bootstrap repetitions; these intervals are descriptive reproducibility outputs rather than definitive inferential intervals.

Table 13: ACTG175 fairness-priority table from the selected baseline.

attribute	FNR gap	FPR gap	ECE gap	recommended	selected
gender_label	0.417	0.040	0.050	yes	yes
homo_label	0.147	0.018	0.033	no	no
drugs_label	0.109	0.036	0.036	no	no
age_group	0.104	0.156	0.034	no	no
race_label	0.040	0.089	0.005	no	no

Table 14: ACTG175 groupwise rates for the selected fairness attribute.

group	N	events	selection	TPR	FNR	FPR	Brier	ECE
Female	70	4	0.314	0.250	0.750	0.318	0.056	0.059
Male	342	21	0.377	0.667	0.333	0.358	0.054	0.009

7.1. ACTG175 mitigation and cross-model benchmarking

The complete exported mitigation tables are included as CSV files in the supplementary data folder. The tables below provide readable summaries of the same evidence.

Table 15: ACTG175 baseline-versus-mitigation comparison, first eight shortlisted candidates.

rank	method	attribute	BA	ROC	FNR gap	FPR gap	Delta BA	FNR gain	FPR gain
1	reweighing	age_group+gender_label	0.595	0.683	0.500	0.095	-0.030	0.250	0.155
2	reweighing	gender_label+race_label	0.620	0.679	0.562	0.102	-0.004	0.237	0.033
3	reweighing	gender_label	0.600	0.679	0.226	0.034	-0.024	0.190	0.006
4	threshold_dp	drugs_label	0.571	0.676	0.022	0.031	-0.053	0.087	0.005
5	reweighing	homo_label	0.599	0.682	0.096	0.043	-0.026	0.051	-0.025
6	threshold_dp	age_group	0.551	0.676	0.104	0.077	-0.073	0	0.079
7	reweighing	gender_label+drugs_label	0.620	0.678	0.750	0.130	-0.004	0	0.101
8	reweighing	gender_label+homo_label	0.599	0.683	0.750	0.351	-0.026	0	0.009

Table 16: ACTG175 held-out 3 by 3 model-mitigation benchmark.

rank	model	mitigation	BA	ROC	gap	Delta BA	gap gain	joint gain
1	ElasticNetLogistic	ThresholdOptimizer-DP	0.646	0.710	0.151	0.046	0.190	yes
2	ExtraTrees	ThresholdOptimizer-DP	0.540	0.568	0.199	-0.060	0.142	no
3	ExtraTrees	ThresholdOptimizer-EO	0.551	0.568	0.210	-0.049	0.131	no
4	ExtraTrees	Reweighing	0.537	0.564	0.298	-0.063	0.043	no
5	LogisticRegression	Reweighing	0.600	0.700	0.341	0	0.000	no
6	LogisticRegression	ThresholdOptimizer-DP	0.634	0.701	0.369	0.034	-0.028	no
7	ElasticNetLogistic	ThresholdOptimizer-EO	0.606	0.710	0.407	0.006	-0.066	no
8	ElasticNetLogistic	Reweighing	0.606	0.710	0.465	0.006	-0.124	no
9	LogisticRegression	ThresholdOptimizer-EO	0.647	0.701	0.724	0.047	-0.383	no

Table 17: ACTG175 true global top-6 held-out comparison.

rank	attribute	model	mitigation	BA	ROC	gap	Delta BA	gap gain
1	age_group	RandomForest	threshold_dp	0.582	0.651	0.180	-0.032	0.091
2	gender_label+race_label	RandomForest	threshold_eo	0.628	0.710	1.089	-0.017	-0.133
3	age_group+race_label	ElasticNetLogistic	threshold_eo	0.624	0.745	0.677	-0.037	-0.080
4	age_group+race_label	LogisticRegression	threshold_dp	0.633	0.726	0.828	-0.027	-0.231
5	age_group+gender_label	LogisticRegression	reweighing	0.637	0.716	0.946	-0.034	0.357
6	gender_label+drugs_label	ElasticNetLogistic	reweighing	0.611	0.732	0.444	-0.005	-0.066

8. Stroke-risk execution outputs

The stroke-risk outputs are reproduced from the exported pipeline artefacts in the last revision package. The final execution selected XGBoost with a validation-selected threshold of 0.06.

Table 18: Stroke-risk model training configuration summary.

model	train N	events	features	CV score	val thr
LogisticRegression	3270	159	6	0.852	0.050
ElasticNetLogistic	3270	159	6	0.853	0.040
RandomForest	3270	159	6	0.849	0.050
ExtraTrees	3270	159	6	0.840	0.050
HistGradientBoosting	3270	159	6	0.845	0.050
SVC_RBF	3270	159	6	0.851	0.040
XGBoost	3270	159	6	0.851	0.060

Table 19: Stroke-risk baseline model performance and fairness summary.

model	thr	BA	ROC	AP	recall	precision	F1	FPR	FNR
XGBoost	0.060	0.773	0.849	0.282	0.820	0.134	0.230	0.274	0.180
RandomForest	0.050	0.767	0.840	0.285	0.800	0.134	0.229	0.266	0.200
HistGradientBoosting	0.050	0.766	0.832	0.203	0.820	0.128	0.222	0.287	0.180
ExtraTrees	0.050	0.765	0.811	0.206	0.800	0.132	0.227	0.271	0.200
SVC_RBF	0.040	0.759	0.839	0.227	0.820	0.122	0.213	0.302	0.180
LogisticRegression	0.050	0.752	0.839	0.241	0.800	0.122	0.212	0.295	0.200
ElasticNetLogistic	0.040	0.749	0.841	0.254	0.840	0.112	0.198	0.343	0.160

Table 20: Stroke-risk top model-mitigation combinations.

rank	model	family	method	attrs	gap reduction	mean Delta BA	min Delta BA	recommended
1	RandomForest	post-processing	Fairlearn DP	7	0.394	-0.002	-0.039	yes
2	RandomForest	post-processing	Group DP	7	0.371	0.005	-0.003	yes
3	RandomForest	intra-processing	Support-aware search	7	0.366	0.003	-0.009	yes
4	RandomForest	post-processing	Group EO	7	0.366	0.003	-0.009	yes
5	RandomForest	post-processing	Group EOpp	7	0.331	-0.003	-0.021	yes
6	RandomForest	post-processing	Group BA	7	0.327	0.004	-0.009	yes
7	LogisticRegression	post-processing	Group DP	7	0.230	0.013	0.007	yes
8	LogisticRegression	intra-processing	Support-aware search	7	0.229	0.005	-0.004	yes

Table 19 summarizes the first six rows of the exported statistical-evidence file. The rows shown correspond to the ElasticNet logistic model with the Fairlearn Exponentiated Gradient demographic-parity mitigation; the complete table is provided as CSV.

Table 21: Stroke-risk statistical evidence table, first six rows.

attribute	metric	Delta	CI 95%	p	improved
Residence	Combined gap	-0.165	[-0.391, -0.013]	0.002	yes
Residence	FNR gap	-0.106	[-0.323, -0.006]	0.016	yes
Residence	FPR gap	-0.059	[-0.118, -0.005]	0.002	yes
Residence x work	Combined gap	-1.632	[-0.758, -0.241]	0.002	yes
Residence x work	FNR gap	-1	[-1.000, -0.150]	0.002	yes
Residence x work	FPR gap	-0.632	[-0.746, -0.560]	0.002	yes

9. Selected-baseline and decision-registry crosswalk

Table 22: Selected-baseline performance crosswalk aligned with the revised manuscript.

Dataset	Model	Thr	BA	ROC-AUC	AP	Recall	Precision	F1	FPR	FNR	ECE	Brier
ACTG175	Logistic regression	0.070	0.624 (0.552-0.651)	0.710 (0.608-0.787)	0.320	0.600	0.099	0.170	0.351	0.400	0.014	0.054
Stroke-risk	XGBoost	0.060	0.773	0.849	0.282	0.820	0.134	0.230	0.274	0.180	0.009	0.041

Table 23: Final decision-registry crosswalk used for the last revision.

Dataset	Decision item	Selected option / caveat
ACTG175	Endpoint	Death by day 365; one-year mortality prediction; cid not used.
ACTG175	Selected model	Logistic regression, isotonic calibration, threshold 0.07.
ACTG175	Fairness priority	gender_label.
ACTG175	Prospective caveat	Treatment arm was not evaluated in a treatment-excluded sensitivity analysis; prospective pre-treatment use would require exclusion or separate analysis.
Stroke-risk	Endpoint	stroke = 1.
Stroke-risk	Selected model	XGBoost, threshold 0.06.
Stroke-risk	Fairness priority	ever_married x Residence_type x work_type x age_group intersection.

10. Machine-readable file indexes

The following tables list only the first entries of each exported file index for readability. The complete indexes and all CSV outputs are included in the supplementary package.

Table 24: ACTG175 extracted notebook-table index, first twelve entries.

table	short description	rows	columns
1	Notebook output table	27	2
2	Setup summary	11	2
3	Support-threshold options	3	7
4	Split-balance checks (top rows)	12	5
5	Feature-policy summary	4	2
6	Predictors requiring attention under the current policy	10	4
7	Requested enabled models: ('LogisticRegression', 'ElasticNetLogistic'...	5	9
8	Probability-version choice	3	9
9	Held-out baseline metric summary with bootstrap confidence intervals	6	5
10	Fairness-priority table	5	6
11	Groupwise rates for the selected fairness attribute: gender_label	2	9
12	Requested mitigation targets (homo_label, drugs_label, race_label+dru...	88	16

Table 25: Stroke-risk execution table index, first twelve entries.

file	rows	columns
00_runtime_and_input_configuration.csv	87	2
00b_acceleration_status.csv	6	2
01_raw_dataset_overview.csv	4	2
02_raw_variable_schema.csv	12	9
03_raw_data_quality_summary.csv	6	2
04_raw_numeric_summary.csv	7	10
05_raw_categorical_summary.csv	8	4
06_fairness_pipeline_configuration.csv	87	2
06b_acceleration_status.csv	7	2
07_automatic_feature_selection_policy.csv	7	10
08_configured_dataset_overview.csv	14	3
09_cohort_or_target_flow.csv	5	2

11. Machine-readable supplementary layer

The PDF tables are curated for readability. The complete machine-readable material is in the accompanying folders. For ACTG175, `data/actg175_death_notebook_tables/` contains 26 tables extracted from the executed one-year mortality notebook, including model selection, probability selection, fairness priority, groupwise rates, mitigation screening, 3 by 3 benchmarking, global top-6 results and the final decision registry. For the stroke-risk dataset, `data/stroke_execution_tables/` contains the numbered execution artefacts from runtime configuration through final decision registry. The `figures/` folder contains selected execution images for both case studies, including ACTG175 notebook figures and stroke-risk graphical outputs. The package also includes a SHA-256 manifest for integrity checks.

12. Treatment-arm and prospective-use caveat

The ACTG175 case study was interpreted as a retrospective post-randomisation methodological demonstration. The treatment-arm variable is therefore defensible only within that retrospective execution context. No treatment-excluded sensitivity analysis was run in the supplied material. For prospective pre-treatment use, treatment assignment should be excluded or analysed in a separate sensitivity analysis before any translational claim is made.

13. Validation and deployment caveat

This supplementary material is an audit and reproducibility package. It does not convert the two public datasets into clinically deployable models. External validation, calibration review, clinical threshold justification, ethics and governance review, workflow integration assessment and prospective monitoring remain necessary before real-world clinical use.
