# Supplementary material for: From Biomedical Datasets to Fairness-Aware Recommendations: An Integrated Data Orchestration Pipeline for Binary Clinical Predictions
Source: Comput Struct Biotechnol J. 2026 Sep 3;35(2):0215. doi: 10.34133/csbj.0215 (PMC13538956; doi:10.34133/csbj.0215)
Supplement: Supplementary file 1 [file csbj.0215.f1.zip › Data_Orchestration_1908.pdf]

From biomedical datasets to fairness-aware recommendations: an integrated data orchestration pipeline for binary clinical predictions

Marta Alberola[a], Pedro Copado[a], Alfredo Vellido[a], Caroline König[a,*]

[a]*Intelligent Data Science and Artificial Intelligence (IDEAI-UPC) Research Centre, Universitat Politècnica de Catalunya - Barcelona Tech (UPC), Jordi Girona 1-3, Barcelona, 08034, Spain*

Abstract

Many problems in biomedicine can be posed as binary classification. When they are addressed using Artificial Intelligence (AI) methods, though, average performance alone does not show whether a dataset is AI-ready, whether the endpoint is clinically valid, or whether errors are unevenly distributed across patient subgroups. This article presents the Fairness-Aware Data Orchestration Pipeline (FADOP), a reusable workflow that analyses biomedical datasets, trains baseline binary classifiers, audits subgroup error patterns, tests mitigation strategies and generates a documented recommendation. Such pipeline is intended for systematic evaluation before clinical translation, not as an automatic deployment tool. Two publicly available case studies illustrate its use: the HIV-related ACTG175 dataset was repurposed from a treatment-comparison trial into a one-year baseline mortality-prediction task, with death by day 365 as the positive class rather than the `cid` AIDS/failure composite endpoint; then a stroke-risk dataset was analyzed as direct event prediction. The case studies show how the same workflow can generate cohort, performance, fairness, mitigation and recommendation evidence across different rare-event clinical datasets.

Keywords: Smart health, Data orchestration, Machine Learning analytical pipeline, Binary classification, Fairness, Bias mitigation, AI-ready datasets, Clinical prediction, Reproducibility

*Corresponding author.
Email address: caroline.leonore.konig@upc.edu (Caroline König)

Highlights

- A clinically-oriented pipeline is proposed for binary biomedical machine-learning classification.
- The workflow evaluates data readiness, endpoint validity, baseline performance, subgroup errors, mitigation options and final recommendations.
- Each pipeline step is linked to the method applied, the output generated and the decision supported.
- Two publicly available case studies show how the same systematic evaluation can be applied to different rare-event datasets.
- Detailed repository and reproducibility artifacts are retained in the supplementary package.

1. Introduction

Artificial intelligence (AI) is increasingly present in clinical research, risk stratification, triage and smart-health decision support. Many clinical prediction tasks are formulated as binary classification: a model receives patient-level information and predicts whether a clinically relevant event will occur or not.

This apparently simple formulation can be clinically fragile. A model may look accurate on average while missing high-risk patients in one subgroup, generating excessive false alerts in another subgroup, or relying on an endpoint that does not match the intended clinical question. Rare outcomes are especially problematic because high overall accuracy can be obtained even when few positive cases are detected. Consequently, dataset preparation, endpoint definition, classification threshold selection and subgroup error analysis are not secondary technical steps. In fact, they determine whether a model can be interpreted and whether its evidence can be responsibly reused.

The current special issue on Data Orchestration in Smart Health is concerned with the route from raw biomedical data to AI-ready resources. This view is highly relevant to clinical machine learning (ML) because the quality, structure, documentation and governance of data often determine the value of the final model. The FAIR principles, datasheets for datasets, model cards

and algorithmic audit frameworks provide complementary foundations for this perspective [1–4].

Fairness is one of the main reasons to make data orchestration systematic. Biomedical datasets can reflect unequal access to care, different measurement intensity, site effects, under-representation of specific populations, historical differences in treatment and proxy outcomes. The well-known example of racial bias in population-health management illustrates that unfairness can arise from the clinical target and measurement process, not only from the model algorithm [5]. Recent biomedical reviews have also shown that fairness assessment remains heterogeneous, that protected attributes are often incompletely documented and that mitigation is not always linked to clinical interpretation [6–8].

The resulting research gap is methodological and translational. Existing fairness toolkits can compute subgroup metrics and apply mitigation methods, but they do not by themselves determine whether a biomedical endpoint is valid, whether features are available at the time of prediction, whether the selected threshold is clinically meaningful, whether subgroup estimates have adequate support or whether a mitigated model should be treated as benchmark evidence rather than deployment evidence. This gap is particularly important in rare-event biomedical prediction, where small positive-class counts make accuracy misleading and where a single missed event can have clinical consequences.

This article presents the Fairness-Aware Data Orchestration Pipeline (FADOP). The objective is not to introduce a new classifier, or a new fairness metric. Instead, we provide a reusable, clinically interpretable and reproducible workflow that analyses a biomedical dataset, evaluates baseline binary prediction models, audits subgroup errors, tests mitigation options and issues a recommendation. The central contribution of the study is, therefore, the data-to-recommendation process. The specific contributions are: (i) an end-to-end data-orchestration workflow for binary biomedical prediction; (ii) an operational fairness audit that preserves false-positive and false-negative components; and (iii) recommendation gates that distinguish retrospective evidence, mitigation candidates and requirements for external validation.

2. Related work

Clinical AI development has increasingly moved from isolated model-building exercises toward broader questions of data quality, model reliability,

subgroup equity and deployment governance, as AI-based decision-support systems are increasingly deployed to support diagnosis, prognostic assessment, and treatment recommendations in real-world clinical settings, including AI-assisted diagnostic tools [9], prognostic prediction systems for conditions such as sepsis [10], and personalized treatment recommendation platforms [11]. In general machine learning, fairness has been formalized through concepts such as individual fairness, demographic parity, equal opportunity and equalized odds [12–17]. These concepts provide a necessary technical vocabulary, but their direct translation into medicine is not straightforward because clinical errors do not have symmetric consequences. A false negative may delay diagnosis, treatment or prevention, whereas a false positive may generate anxiety, unnecessary testing and inefficient allocation of clinical resources. For this reason, in this study fairness is not treated as a single universal parity constraint, but as the absence, or clinically justified reduction, of systematic and unsupported differences in model errors across relevant patient subgroups at the selected clinical operating point.

This operational definition is also motivated by the well-known incompatibility between some fairness criteria when baseline risks differ across groups [18–20]. Such differences are common in biomedical datasets, where age, comorbidity, treatment history, disease severity and social determinants may influence both outcome prevalence and data availability. A clinically useful fairness audit should therefore avoid reducing the evaluation to a single aggregate number. Instead, it should make visible where the model misses events, where it over-alerts, how much subgroup support exists, whether subgroup estimates are stable, and whether mitigation improves fairness without unacceptable loss of clinical utility. This is the perspective adopted in FADOP, where subgroup FPR, FNR and TPR gaps are interpreted together with ROC-AUC, balanced accuracy, recall, F1-score, uncertainty and recommendation gates.

The biomedical fairness literature has shown that bias may enter the AI lifecycle through multiple mechanisms. Systematic reviews and surveys have reported that bias in biomedical AI can arise from cohort construction, sampling imbalance, label noise, missingness, acquisition protocols, institutional practice patterns, disease prevalence, subgroup under-representation and model-selection procedures [6–8]. Correa et al. emphasized that much of the early fair-AI literature in image classification and prediction focused on detecting disparities, while fewer studies proposed complete and clinically actionable methods for reducing them [6]. Yang et al. extended this view by

organizing biomedical debiasing methods into distributional and algorithmic families, including data augmentation, perturbation, reweighting, federated learning, adversarial learning, representation learning and causality-based methods [7]. Liu et al. further highlighted an evidence gap between technical fairness solutions and clinical implementation, noting that AI fairness research remains unevenly distributed across medical fields, bias-relevant attributes and clinician-in-the-loop settings [8]. These reviews support the central motivation of the present work: clinical fairness assessment needs a reproducible workflow that links data readiness, endpoint construction, model evaluation, subgroup auditing and deployment-oriented interpretation.

Medical-imaging fairness studies have reinforced the same conclusion from a different data modality. MEDFAIR showed that fairness benchmarking is sensitive not only to the mitigation algorithm, but also to datasets, sensitive attributes, model-selection criteria and evaluation metrics [21]. Its finding that state-of-the-art mitigation methods do not consistently outperform empirical risk minimization is particularly relevant to FADOP, because it supports our decision to treat mitigation as evidence to be audited rather than as an automatic solution. Similarly, Jones et al. showed that subgroup separability can influence how models learn and express bias: when protected-group information is inferable from the input data, models may learn subgroup-specific mappings even when explicit sensitive variables are removed [22]. This observation is directly relevant to tabular clinical prediction, where age, treatment history, laboratory patterns and clinical variables can act as proxies for sensitive or socially meaningful attributes. Puyol-Antón et al. showed in cardiac MR segmentation that training-data imbalance can produce measurable group differences and that mitigation strategies such as stratified sampling, fair meta-learning and protected-group models may reduce those differences [23]. Li et al. likewise demonstrated that domain-adversarial strategies can be designed to address multiple sensitive features in disease prediction, while also handling class imbalance through AUC-oriented losses [24]. Together, these studies indicate that fairness improvement is task-specific, model-specific and data-specific; this justifies the FADOP choice to compare multiple model families and mitigation strategies under a common audit structure rather than assuming that one debiasing method will generalize.

Recent research places these fairness concerns within a broader biomedical-computing context. One important direction is distributed and privacy-preserving learning: Hoang *et al.* presented APPFLx as an end-to-end secure federated-learning framework for biomedical research in heterogeneous

computing environments [25], while Tajabadi et al. [26] reviewed privacy-preserving decentralized learning approaches for biomedical applications, and Madathil *et al.* [27] surveyed federated learning for healthcare data analytics. The importance of such privacy-preserving approaches is underscored by evidence that ML models can leak sensitive information even when trained on small datasets [28], a particularly critical concern for medical data. Together, these works show that biomedical AI is increasingly expected to operate across institutions, infrastructures and data-governance constraints, rather than within a single centralized dataset. They also highlight that privacy-preserving computation, although essential, addresses only one part of trustworthy AI: models trained in distributed settings still need careful evaluation of data quality, subgroup representation and generalizability.

A second line of research concerns patient-centred data governance and the growing role of patient-generated information. Kim *et al.* proposed a self-sovereign management scheme for personal health records based on personal data stores and decentralized identifiers [29]. Folkvord *et al.* discussed the use of patient-generated health data in value-based healthcare implementation, emphasizing the opportunities and challenges associated with data produced outside traditional clinical encounters [30]. These studies suggest that future biomedical AI systems will increasingly rely on data whose ownership, provenance, completeness and representativeness are heterogeneous. This has direct implications for fairness because patient-generated and self-managed data may improve longitudinal coverage for some populations while introducing access, adherence, device and measurement biases for others.

Synthetic data has also become an important topic in biomedical AI because it may help address privacy restrictions, rare outcomes and limited sample sizes. Pezoulas *et al.* reviewed open-source tools and methods for healthcare synthetic data generation [31]. Their review shows that synthetic data can support methodological development, benchmarking and privacy-aware data sharing, but also that its quality depends on how well the generating process preserves clinically meaningful distributions. Nisevic *et al.* analysed legal and ethical issues related to synthetic data and patient profiling in medicine [32]. Their discussion is particularly relevant to fairness because synthetic data may reproduce, conceal or amplify bias if the source population is already unbalanced or if generated profiles are later treated as if they were direct clinical evidence.

Other studies focus on biomedical data integration, digital twins and advanced computational infrastructures. Cheng *et al.* proposed an instance-level

medical image classification method for text-based retrieval within a Medical Data Integration Center [33]. Their work reflects a broader movement toward integrated biomedical repositories in which images, metadata and textual queries can be connected for retrieval and downstream analysis. Wu and Koelzer discussed generative digital twins as an emerging paradigm for biomedical research [34]. Digital twins extend the idea of clinical prediction toward simulation and individualized representation, but their usefulness depends on the quality, validity and representativeness of the data and assumptions used to construct them. These contributions show that biomedical AI is moving toward more complex systems in which fairness cannot be evaluated only at the final model-output level, but must also be considered in the data structures and computational representations that precede prediction.

Trustworthy clinical AI also requires interpretability and secure deployment. Huang et al. examined the gap between explainable and interpretable deep learning in healthcare natural language processing [35]. Their analysis distinguishes between producing post-hoc explanations and achieving explanations that are meaningful for clinical users, which remains a major challenge in healthcare AI. Rehman *et al.* proposed a blockchain-enhanced lightweight protocol for secure cloud–IoT communication in biomedical healthcare cyber-physical systems [36]. This work illustrates that biomedical AI systems may depend on secure communication across connected devices, cloud services and healthcare infrastructures. Security, explainability and fairness are therefore related dimensions of trustworthiness, although each addresses a different kind of risk.

Taken together, these works describe a biomedical AI landscape shaped by federated learning, decentralized governance, patient-generated data, synthetic data, data integration, digital twins, interpretability and secure cyber-physical infrastructures [25–27, 29–36]. Their common conclusion is that biomedical AI cannot be evaluated only as an isolated algorithmic object. It must be understood as part of a larger data and decision ecosystem in which privacy, provenance, interoperability, clinical meaning, interpretability, security and subgroup effects all influence whether a system can be responsibly translated.

The FADOP system presented in this study can be seen as part of a broader transition from isolated biomedical AI models toward governed, interoperable and deployment-aware smart-health infrastructures. Many of the aforementioned studies address how data can be distributed, protected, generated, integrated, interpreted and transmitted. The gap addressed by FADOP is different but connected: how to convert a biomedical dataset into

an auditable fairness-aware recommendation for a binary clinical prediction task. This includes checking whether the dataset is AI-ready, whether the endpoint matches the clinical question, whether baseline models provide useful discrimination at a meaningful threshold, whether subgroup errors are acceptable, whether mitigation improves the fairness–utility balance, and whether the final evidence supports only benchmarking, further validation, or cautious translational consideration.

Several existing software toolkits already support fairness measurement and mitigation, including AI Fairness 360, Aequitas and Fairlearn [37–40]. These tools are valuable, but a fairness toolkit is not the same as a clinically-grounded data-orchestration framework. Biomedical prediction also requires decisions before and after the fairness method is applied: the endpoint must correspond to the clinical question, variables must be available at prediction time, missingness and data quality must be documented, model families must be justified, thresholds must be clinically interpretable and subgroup estimates must have adequate support. Leakage control is particularly important because clinical datasets may contain follow-up duration, censoring fields, post-baseline measurements, treatment variables or outcome-derived information that would not be available at the intended time of prediction. If such variables are not excluded, model performance can be artificially inflated and subgroup audits can become misleading.

Within this broader literature, the present article focuses on fairness-aware binary clinical prediction from structured biomedical datasets. The specific emphasis is not placed on proposing a new fairness definition or a new classifier, but on organizing the steps that connect dataset inspection, endpoint definition, model training, thresholded evaluation, subgroup-error auditing, mitigation comparison and recommendation reporting.

Methodologically, FADOP follows standard statistical learning practice while extending it with fairness-aware decision logic. Model families are compared rather than assumed, cross-validation is used for hyperparameter selection, ranking metrics are separated from thresholded metrics, and uncertainty or paired comparisons are used to avoid over-interpreting single-split improvements [41–45]. The novelty of FADOP is therefore not the invention of a new classifier or a new fairness metric. Instead, it lies in integrating established statistical learning, clinical endpoint construction, AI-readiness assessment, subgroup-error auditing, mitigation comparison and recommendation gates into one reusable workflow for fairness-aware biomedical binary prediction.

3. FADOP framework

3.1. Novelty and purpose of the pipeline

The novelty of FADOP lies in the integration of analysis, mitigation and recommendation into a single reproducible workflow for biomedical binary classification. Many studies report a predictive model and, at most, a limited set of subgroup metrics. In contrast, this pipeline treats the complete route from dataset to recommendation as the methodological contribution.

The pipeline addresses five practical needs that are common when a biomedical dataset is reused for binary ML-based prediction. First, it evaluates whether the dataset is sufficiently described, complete and coherent for modelling. This includes the number of records, variable types, missingness, duplicated rows and the availability of candidate predictors before the prediction time. Second, it checks whether the outcome and, when relevant, the prediction horizon are clinically meaningful. This is necessary because a technically valid column may still be a poor clinical label if it mixes events, follow-up artifacts or post-outcome information. Third, it compares several baseline models and selects an operating threshold suited to rare-event prediction, where the default 0.5 threshold may miss many true events. Fourth, it audits subgroup errors, especially false negatives and false positives, to identify whether the model behaves differently across clinically- or ethically-relevant groups. Fifth, it tests mitigation options and translates the results into a recommendation that can be reviewed jointly by clinicians, data scientists and governance teams.

This structure is important because mitigation alone is not enough. A mitigation method may reduce one numerical fairness gap while simultaneously reducing sensitivity, increasing false alerts, worsening calibration or relying on a subgroup represented by only a few patients. For this reason, FADOP does not automatically declare that a model is fair after mitigation. It separates three questions: whether the data and endpoint are suitable, whether the model has acceptable clinical utility and whether any fairness improvement is supported by enough evidence. Therefore, the final output is a decision category rather than a simple approval: return to data preparation, use the model only as a benchmark, validate a mitigation candidate externally, or consider a carefully governed clinical-evaluation pathway only when external validation, calibration review and clinical threshold justification are available.

Table 1 summarises the intended distinction between FADOP and closely related frameworks. The comparison is functional rather than competitive:

FADOP uses existing ML and fairness libraries where appropriate, but adds the clinical-data orchestration and recommendation layer around them.

Table 1: Functional positioning of FADOP relative to common fairness and medical-AI benchmarking frameworks. A check mark indicates a primary design feature; a dash indicates that the feature is not the central purpose of the framework.

**Feature**	**FADOP**	**Fairlearn**	**AI Fairness 360**	**MEDFAIR**
Tabular biomedical binary-prediction workflow	✓	Partial	Partial	–
Endpoint validity and prediction-horizon checks	✓	–	–	–
Feature availability and leakage-control policy	✓	–	–	–
Baseline model comparison and clinical threshold selection	✓	Partial	Partial	Partial
Subgroup support warnings before interpreting fairness gaps	✓	Partial	Partial	Partial
Pre-, in- and post-processing mitigation options	✓	✓	✓	✓
Recommendation gates for data revision, benchmark evidence and external validation	✓	–	–	–
Primary benchmark domain	Clinical tabular prediction	General ML	General ML	Medical imaging

3.2. Pipeline overview

The main workflow is shown in Figure 1. It starts with a clinical binary-prediction question and ends with a documented recommendation. The workflow has been designed for datasets in which the outcome can be represented as event versus no-event, including direct binary targets and fixed-horizon transformations of follow-up data. The purpose is not only to train a model, but also to show how the data, endpoint, model threshold, subgroup errors and mitigation options justify the final recommendation.

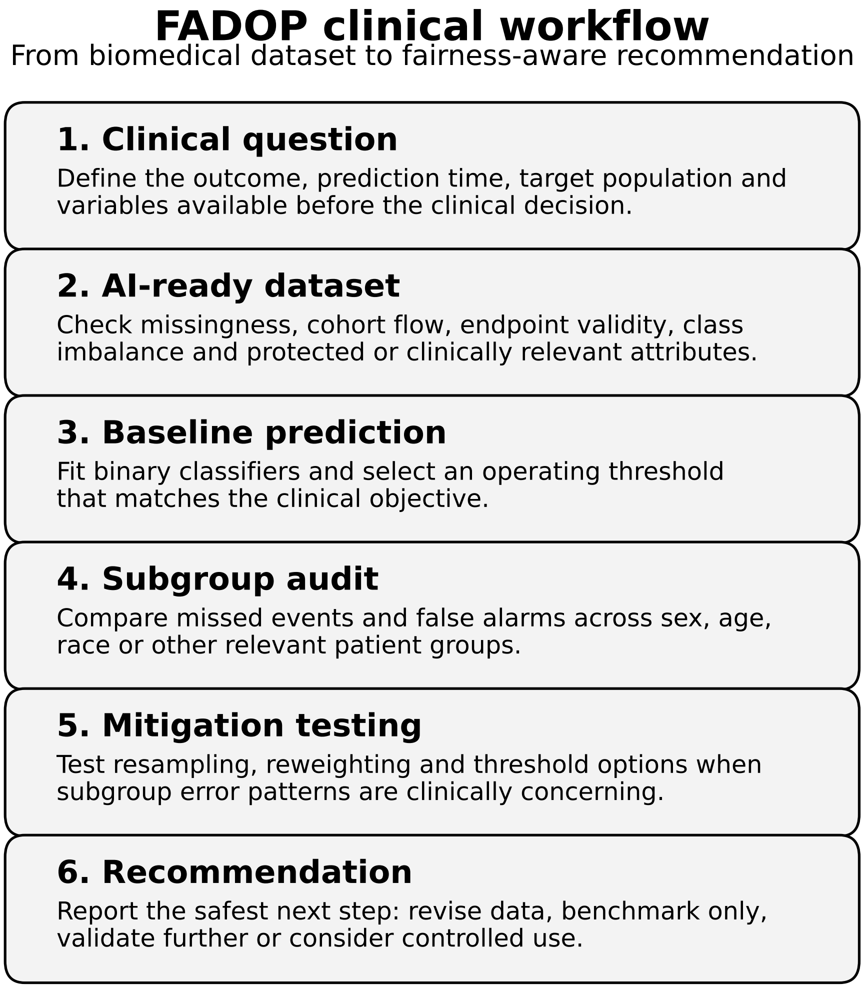

Figure 1: Clinical interpretation of FADOP. The workflow translates a biomedical binary-prediction problem into an evidence package containing data-readiness checks, baseline performance, subgroup error analysis, mitigation assessment and a final recommendation.

3.3. Statistical analyses performed at each pipeline step

The statistical workflow is organised as a sequence of decision points rather than as a single model-fitting routine. Each step generates an output that answers a practical question for the analyst or clinical reviewer: whether

the data can be used, whether the target is clinically coherent, whether the baseline model is useful, whether errors are unevenly distributed, whether mitigation improves the situation and whether the evidence is strong enough to support the next stage of evaluation.

The first step is raw-data intake and AI-readiness assessment. The pipeline counts records and variables, identifies duplicate rows, classifies variables as numerical, categorical or binary, and summarises missingness. A missing entry is defined as an empty cell, a software-recognised null value or a configured missing-value token that cannot be interpreted as a valid measurement or category. Missingness is evaluated both at feature level, to identify variables with high missing rates, and at record level, to identify patients with incomplete information. The pipeline reports missingness descriptively; it does not infer whether the missing-data mechanism is missing completely at random (MCAR), missing at random (MAR) or missing not at random (MNAR), because this requires clinical and data-generation knowledge beyond the table itself. Numerical variables are described through distributional summaries, while categorical variables are described through frequency tables. Data quality is also broader than missingness: the readiness stage flags duplicate records, implausible or out-of-range values when configured, corrupted category encodings, identifier-like fields, very rare categories, noisy measurements and possible label or endpoint inconsistencies. These checks influence the readiness score as warnings or stop conditions depending on severity. Sample size is assessed through total records, event counts, split-level positive cases and subgroup support, because the minimum useful sample depends on event prevalence, model complexity and the number of audited subgroups. The user decision at this stage is whether the dataset can proceed to endpoint construction or whether data cleaning, documentation, larger sample collection or clinical endpoint review are required.

The second step defines the endpoint and the analytic cohort. When a direct binary outcome is available, the positive class is explicitly encoded. When the dataset contains follow-up information, a fixed-horizon endpoint is constructed from event status and follow-up time. Records censored before the selected horizon are excluded because the event status at that horizon is unknown. Event rates are then reported overall and by relevant subgroups. This step prevents the model from being trained on an ambiguous label and forces the prediction task to be stated in clinical terms.

The third step separates prediction variables from protected or clinically sensitive attributes. Sensitive attributes are retained for auditing, but are not

used as predictors unless this is explicitly justified in the configuration. Age can also be grouped for interpretation when clinically useful. The output is a sensitive-attribute summary with subgroup sizes and event rates. The user decision is whether each variable should be used for prediction, reserved for fairness auditing, or excluded because of governance concerns.

The fourth step performs feature screening and leakage control. Leakage control means verifying that candidate predictors are truly available at the moment when the model would be used. In retrospective clinical datasets, variables recorded after baseline, follow-up duration, censoring indicators, post-event procedures, treatment changes after the outcome, discharge variables or outcome-derived fields can accidentally reveal the answer to the model. The pipeline therefore flags identifier-like fields, target-derived names, high-cardinality variables, near-duplicate variables and variables with unusually strong target association. Near duplicates are removed when the absolute Pearson correlation with an already retained numerical variable is at least 0.995. Association with the target is summarised using absolute Pearson correlation for continuous variables and Cramer's V for categorical or discrete variables [46–48]. Proxy behaviour against sensitive attributes is reviewed using the same logic. The output is a feature-policy table explaining which variables are retained, excluded or flagged for manual review.

The fifth step creates training, validation and test partitions using a fixed random seed. Stratification is used to preserve the event distribution and, where feasible, subgroup composition. The pipeline reports event and non-event counts in each partition. This is particularly important in rare-event datasets because a split with too few positive cases can produce unstable estimates. As a practical guideline, FADOP treats the number of events in the validation and test sets, the events per candidate predictor and the minimum subgroup size as interpretation constraints rather than universal cut-offs. If the positive class or an audited subgroup has very few cases, the pipeline demotes conclusions to exploratory evidence even when a point estimate appears favourable. The user decision is whether the downstream evidence can be interpreted as a model comparison, or only as exploratory analysis [43].

The sixth step trains and compares baseline classifiers. Numerical variables are median-imputed and standardised; categorical variables are most-frequent imputed and one-hot encoded. The model families are trained for complementary reasons, so that the benchmark does not depend on one modelling assumption, and they represent established statistical-learning families imple-

mented in common Python ML workflows [41, 42, 49]. Logistic regression is trained as a transparent linear baseline that estimates an additive association between predictors and the event log-odds [50]. It is chosen because it is clinically interpretable, relatively stable in small datasets and useful as a reference for more flexible methods. Elastic-net logistic regression is trained as a regularised linear model that combines variable shrinkage and variable selection [51]. It is chosen because biomedical tables can contain correlated predictors, sparse one-hot categories and limited event counts, and regularisation reduces overfitting risk. Random forests are trained as bagged ensembles of decision trees that can capture nonlinear effects and predictor interactions without requiring explicit interaction terms [52]. They are chosen because they are robust baselines for mixed clinical variables and can perform well when the relationship between predictors and outcome is not linear. Extra Trees are trained as more randomized tree ensembles that increase diversity among trees by using random split choices [53]. They are chosen because this additional randomness can reduce variance and provide a useful contrast to conventional random forests. Histogram gradient boosting is trained as a boosted tree model that sequentially corrects previous errors using discretised feature bins [54]. It is chosen because it is computationally efficient and often strong for tabular clinical datasets with nonlinear risk patterns. RBF-SVMs are trained as margin-based classifiers with a radial basis kernel [55]. They are chosen because they test whether smooth nonlinear decision boundaries improve ranking when the number of predictors is moderate. XGBoost is trained as a regularised gradient-boosted tree ensemble [56]. It is chosen because it is a high-performing tabular-data model and provides a strong benchmark for nonlinear interactions and imbalanced risk prediction. Rather than immediately assigning discrete binary labels ($\hat{y} \in \{0, 1\}$), these binary classifiers first output continuous risk scores representing estimated probabilities $\hat{p} = P(Y = 1 \mid X)$. Linear and boosted-tree models (e.g., Logistic Regression, XGBoost, Histogram Gradient Boosting) output calibrated probabilities via logistic transformations or log-loss optimization [50, 56], whereas RBF-SVMs map margin distances to continuous probabilities using Platt scaling [57]. Preserving these continuous risk scores before thresholding is essential for downstream operating point optimization and subgroup disparity analysis.

Hyperparameters are selected with stratified cross-validation using ROC-AUC as the ranking metric [43, 58]. Deep neural networks and transformer architectures were not included in the reported baseline because both case

studies are small-to-moderate tabular rare-event datasets with no imaging, free-text or longitudinal sequence input. In this setting, conventional statistical learning and tree-ensemble models provide stronger interpretability, lower sample-complexity and more stable benchmarking. This step avoids choosing one algorithm a priori and produces a benchmark against which mitigation can be judged.

The seventh step selects the operating threshold on the validation set to convert continuous predictions into binary clinical decisions. Candidate thresholds τ are systematically evaluated across the range $[0.01, 0.80]$, and the selected threshold maximizes balanced accuracy, with secondary tie-breakers based on F_1-score and selection rate. Explicitly tuning τ is critical because standard default cutoffs (e.g., $\tau = 0.5$) are fundamentally inadequate in rare-event clinical scenarios; under severe class imbalance, a 0.5 threshold forces models to optimize for overall accuracy, resulting in trivial majority-class predictions that yield high specificity but miss critical true events [59, 60]. Varying the decision threshold directly dictates the clinical trade-off between sensitivity (recall), false-positive rate (FPR), and false-negative rate (FNR). Because the clinical costs of missing a rare condition (a false negative) often vastly outweigh those of a false positive, optimizing τ aligns model predictions with asymmetric decision risks and clinically relevant threshold preferences [61]. Finally, on the held-out test set, the pipeline evaluates performance at this chosen operating point alongside threshold-agnostic and calibration metrics, reporting accuracy, balanced accuracy, precision, recall, F_1-score, ROC-AUC, average precision, Brier score, expected calibration error, false-positive rate, false-negative rate, and selection rate [62–65].

The eighth step audits subgroup fairness at the selected threshold. For each audited attribute and for supported intersections, the pipeline computes subgroup confusion matrices, event counts, true-positive rates, false-negative rates, true-negative rates, false-positive rates, selection rates and accuracies. The main operational disparity measure is the combined false-positive-rate and false-negative-rate gap. The combined gap is used only as a screening signal so that many attributes can be ranked in a single audit table; the false-positive and false-negative components remain separately reported because they have different clinical meanings. A large false-negative-rate gap means that events are missed more often in some groups. A large false-positive-rate gap means that some groups receive more unnecessary alerts. Selection rate is also reported because it approximates how many patients would be flagged for follow-up and therefore affects workload and resource allocation. Very

small intersectional groups are not treated as robust fairness estimates; they are flagged as support warnings. Because many attributes and intersections can be screened, the audit is interpreted as hypothesis-generating rather than confirmatory. The pipeline does not treat every large observed gap as statistically definitive; it uses support warnings, bootstrap evidence and clinical review to limit multiplicity-driven over-interpretation.

The ninth step tests mitigation strategies when relevant disparities are detected. Pre-processing methods modify the training data before model fitting. Reweighing changes the contribution of records during learning so that under-represented classes or groups have more influence on the fitted model [66]. Random oversampling duplicates minority-class records, and can be applied when event cases are rare but the analyst wants to avoid changing the learning algorithm [59]. SMOTE creates synthetic minority-class samples by interpolating between neighbouring minority examples in feature space [67]. It can be applied after numeric preprocessing when the minority class is too small for stable learning, but it must be used carefully because synthetic records should not be interpreted as real patients. SMOTEN is a nominal-feature variant of the same general idea, designed for categorical predictors where simple numeric interpolation would create invalid category values [68]. SMOTEENN combines SMOTE-style oversampling with Edited Nearest Neighbour cleaning, so it both adds minority examples and removes ambiguous or noisy boundary cases [68, 69]. These resampling methods can be useful when class imbalance, subgroup imbalance or noisy class boundaries contribute to error gaps, but they are not automatically appropriate if the dataset is very small, if categorical structure is poorly encoded or if synthetic examples would be clinically implausible. In-processing methods modify the learning procedure when compatible with the selected model family, for example by adding fairness constraints or adversarial objectives [24, 70]. Post-processing methods modify the decision rule after training, for example through group-specific thresholds or Fairlearn threshold optimisation for demographic parity or equalized odds [13, 39, 40]. Mitigation methods are evaluated comparatively, because the best family depends on the dataset, the model and the subgroup pattern [7, 21, 71].

The final step applies uncertainty analysis and recommendation gates. Bootstrap resampling of the test set estimates confidence intervals and empirical p-values for changes in false-positive-rate gap, false-negative-rate gap, combined error gap and balanced accuracy [44]. Exact McNemar tests are also calculated when paired subgroup error changes can be compared before and

after mitigation [45]. A performance guard is then applied: a mitigation candidate is not recommended if the improvement in subgroup error is obtained at the cost of an unacceptable reduction in balanced accuracy. In the reported experiments, the configured tolerance was a maximum balanced-accuracy decrease of 0.05. Clinically, because balanced accuracy averages sensitivity and specificity, a 0.05 decrease could correspond approximately to a 0.10 loss in sensitivity if all the loss falls on event detection, which at the observed 4.9–6.0% event rates would mean roughly 0.5–0.6 additional missed events per 100 assessed patients; if the loss falls on specificity, it would correspond to about 9–10 additional false alerts per 100 assessed patients. The value is therefore a transparent default guard rather than a universal clinical rule, that can also be modified and selected by the user. The final output is not deployment approval, but a decision category: revise the dataset, retain the result as benchmark evidence, validate a mitigation candidate externally, or proceed only under a governed clinical-evaluation pathway.

The main statistical quantities are defined in clinically interpretable terms. Balanced accuracy is used because both event and non-event recognition matter in imbalanced clinical prediction:

$$\text{Balanced accuracy} = \frac{\text{Sensitivity} + \text{Specificity}}{2}. \tag{1}$$

For subgroup auditing, false-positive and false-negative rates are computed from the confusion matrix. For an audited attribute A, the operational combined error gap is:

$$\text{Combined gap}(A) = \left[\max_g \text{FPR}_g - \min_g \text{FPR}_g\right] + \left[\max_g \text{FNR}_g - \min_g \text{FNR}_g\right], \tag{2}$$

where g indexes the groups within attribute A. This definition is used as a practical safety-oriented screen because it summarizes both unnecessary alerts and missed events. It is not presented as a universal ethical definition of fairness, and FADOP therefore retains the FPR and FNR components in the audit tables and figures.

3.4. Software organization

The technical architecture is summarized in Figure 2 and in Table 2. Dataset-specific choices are separated from reusable code, so the same pipeline can be rerun on different biomedical datasets while preserving a common output structure.

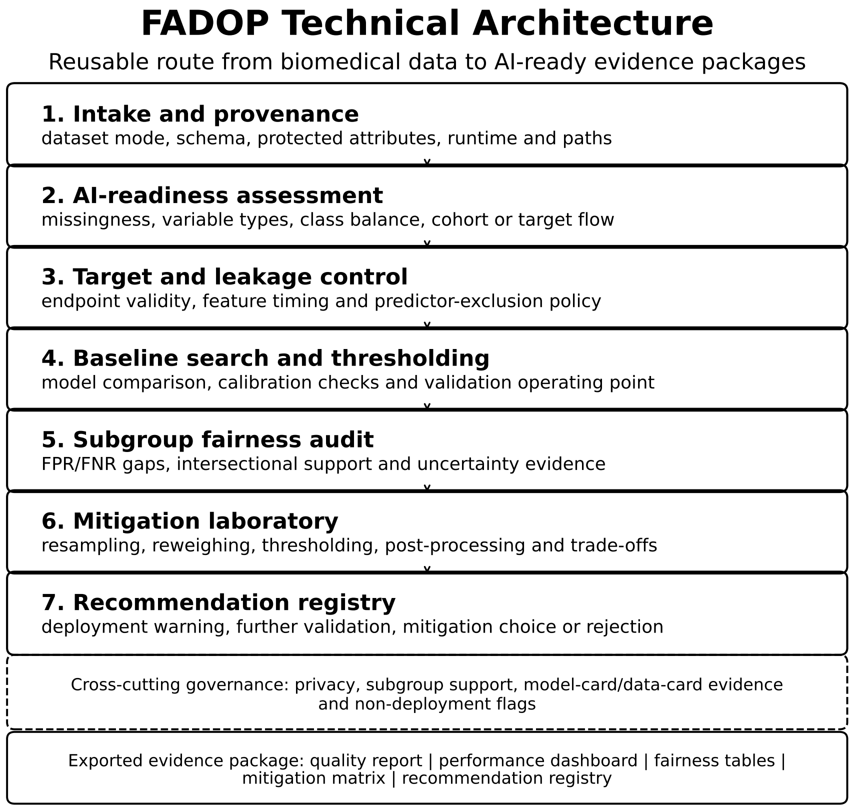

Figure 2: FADOP Technical Architecture. Raw biomedical data are converted into AI-ready evidence through linked stages for schema inspection, target construction, feature governance, modelling, fairness audit, mitigation and recommendation.

Table 2: Reusable software layers in the GitHub-ready implementation.

Layer	Purpose	Typical outputs
Configuration	Stores dataset mode, target, protected attributes, candidate predictors, missing-data policy and output paths.	Reproducible run configuration and analysis manifest.
Runtime control	Handles seeds, acceleration checks, logging and controlled execution order.	Runtime report, acceleration status and progress logs.
Data preparation	Reads data, checks schema, summarizes missingness, constructs binary endpoints and creates train-validation-test splits.	Dataset overview, cohort flow, feature policy and split summaries.
Modeling	Fits baseline binary classifiers and evaluates validation thresholds.	Model comparison table, threshold sweep and selected operating point.
Fairness evaluation	Computes subgroup performance, false-positive and false-negative patterns and intersectional support.	Fairness-by-group tables, subgroup support audit and gap summaries.
Mitigation	Tests pre-processing, in-processing and post-processing options when fairness concerns are observed.	Mitigation rankings, group thresholds and statistical evidence tables.
Reporting	Produces figures, recommendation registry, model-card-style summaries and artifact manifest.	Decision registry, visual summaries and reproducibility package.

3.5. Mitigation and recommendation gates

The mitigation stage is followed by recommendation gates summarized in Figure 3. In this article, a gate is a checkpoint that prevents a technically attractive result from being interpreted as clinically ready before the necessary conditions have been reviewed. The gates translate the pipeline outputs into

a small number of action categories. They are intended to support discussion between clinical users, data scientists and governance teams, not to replace expert review.

The first gate is the data-readiness gate. It asks whether the endpoint, prediction horizon, cohort construction, missing-data profile and feature policy are acceptable. If the label is unclear, if relevant variables are not documented, or if leakage cannot be ruled out, the appropriate recommendation is to return to data preparation rather than to optimise the model.

The second gate is the performance guard. This is the rule that a fairness intervention must not make the model clinically unusable. In the current implementation, the guard compares balanced accuracy before and after mitigation and rejects or demotes a candidate when the balanced-accuracy loss exceeds the configured tolerance. In the reported experiments this tolerance was set to a value of 0.05. The guard is necessary because a mitigation method can reduce a subgroup gap simply by making predictions less informative. Sensitivity, false-negative rates, precision and calibration still require clinical review, but balanced accuracy provides a transparent first guard against unacceptable utility loss.

The third gate is the fairness-and-support gate. It evaluates whether subgroup error gaps are reduced, whether false-negative and false-positive changes move in a clinically acceptable direction, and whether the relevant subgroups contain enough patients to support interpretation. A mitigation candidate with apparent improvement in a group of one or two patients is not treated as robust evidence. It is treated as a signal that more data, subgroup monitoring or data harmonization may be needed.

The fourth gate is the governance gate. Even when data readiness, performance and fairness evidence are favorable, the output is still not one of deployment approval. Operational use would require external validation, calibration assessment, clinical threshold justification, monitoring plans, privacy and ethics review, and human oversight. The recommendation layer therefore distinguishes four possible outcomes: revise the dataset, retain the model only as benchmark evidence, validate a mitigation candidate externally, or consider a governed clinical-evaluation pathway.

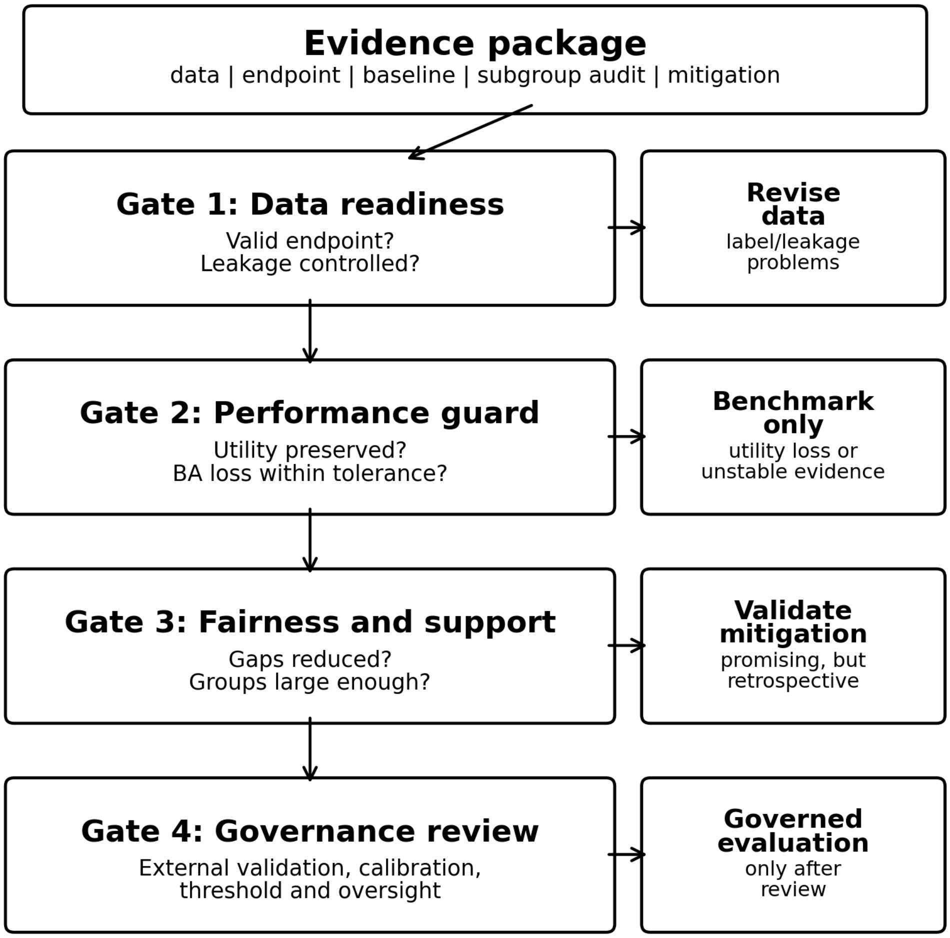

Figure 3: Recommendation gates used by FADOP. A gate is a decision checkpoint that converts data-readiness, performance, fairness, support and governance evidence into an action category. The pipeline distinguishes data revision, benchmark-only evidence, mitigation validation and potential governed evaluation. Even a successful mitigation candidate requires external validation, calibration review, clinical threshold justification and governance review before operational use.

4. Case Study Experiments

Two publicly available datasets were used to demonstrate the proposed workflow. Their purpose was to test whether the same pipeline could analyze different biomedical binary-prediction problems and produce comparable evidence packages.

4.1. Datasets and endpoint construction

The first case study used ACTG175, a clinical-trial dataset originally designed to compare antiretroviral treatment outcomes in HIV-infected adults with intermediate CD4 counts [72, 73]. Its original purpose was therapeutic and comparative: different treatment arms were evaluated to determine whether clinical outcomes differed between regimens. In this article, the dataset was reused with a different and single predictive objective. Treatment comparison was not the aim of the analysis, and no causal conclusion about treatment efficacy was attempted. Instead, the task was redefined as a fixed-horizon baseline prediction problem. Operationally, the positive class was death observed by day 365. The ACTG175 execution constructed the modelling target from the death-specific one-year status and did not use `cid` as the endpoint variable. Records with observable alive/death status through day 365 were retained; participants whose one-year mortality status could not be determined because follow-up ended before the horizon were excluded from the analytic cohort. Participants known to be alive at day 365 were treated as non-events. Therefore, the ACTG175 case study should be interpreted as one-year mortality prediction, not as an AIDS/failure, treatment-failure, or clinical-progression composite endpoint.

For the second case study, a public Kaggle stroke-risk dataset [74] was used. The analysed upload was `ranaghulamnabi/stroke-risk-dataset`, accessed from the Kaggle URL recorded in the reference list on 5 June 2026. This dataset was analyzed as a standard binary classification task, with stroke as the positive class. The two datasets therefore represented rare-event prediction problems, but with different endpoint-construction requirements: ACTG175 required a death-specific fixed-horizon target, whereas the stroke-risk dataset had a direct binary target.

Both datasets are summarily described in Table 3.

Table 3: AI-readiness and cohort-construction outputs.

Output	ACTG175	Stroke-risk dataset
Original role of the dataset	Clinical trial designed to compare antiretroviral treatment outcomes.	Public tabular dataset for stroke-risk classification.
Pipeline task	Secondary baseline prediction of one-year mortality; death by day 365 was the positive class and treatment comparison was not used as the target.	Direct prediction of stroke versus no stroke.
Raw records	2,139	5,110
Analytic records	2,058 after excluding 81 records without observable one-year mortality status.	5,110 records retained for the direct binary target.
Positive events	123 one-year deaths; 5.98% event rate.	249 stroke events; 4.87% event rate.
Predictor policy	13 baseline predictors retained under the configured `keep_all` feature policy for the mortality analysis.	6 predictors after excluding targets, sensitive attributes and leakage-prone fields.
Audit attributes	Age group, gender, race, homosexual-activity label and drug-use label.	Gender, marital status, residence type, work type and age group.

The cohort-flow table supported two decisions. In ACTG175, the exclusion of 81 records was necessary because these participants did not have observable one-year mortality status. In the stroke-risk dataset, no rows were excluded for missing target values, so the full raw cohort was retained. Both datasets had rare positive classes, which justified the use of balanced accuracy, sensitivity, false-negative rates and threshold selection rather than overall accuracy alone.

4.2. AI-readiness and feature-screening decisions

The data-quality step found no missing cells or duplicate rows in the ACTG175 source table. The stroke-risk source table contained 201 missing cells and no duplicate rows; the missingness was handled inside the preprocessing workflow. In both case studies, missingness was counted as blank,

null or software-recognized missing values at feature and record level. The workflow did not infer MCAR, MAR or MNAR status, but it preserved the missingness summary as a readiness artifact for clinical review. The feature-policy step then converted these observations into explicit modelling decisions. In the ACTG175 mortality execution, all 13 candidate baseline predictors were retained under the configured `keep_all` policy, while leakage or target-derived fields such as one-year alive/death status and post-baseline follow-up variables were excluded from the predictor set. Randomized treatment arm was retained only because the demonstration is a retrospective post-randomization baseline-risk analysis in which allocation is known at baseline. For a prospective pre-treatment triage use case, treatment arm would be unavailable and should be excluded or handled through a separate treatment-policy analysis. A treatment-excluded sensitivity run was not part of the supplied final notebook; therefore, the ACTG175 case study is not presented as a prospective pre-treatment risk model. In the stroke-risk dataset, the identifier column was dropped and six clinical or behavioral predictors were retained. All these decisions are summarized in Table 4.

Table 4: Examples of statistical feature-screening outputs used to justify inclusion, exclusion and review decisions. Association and proxy values are screening statistics, not causal effects.

**Screening item**	**ACTG175**	**Stroke-risk dataset**
Data-quality decision	No missing cells or duplicate rows; no correction was required before one-year mortality endpoint construction.	201 missing cells were detected; model preprocessing therefore retained median imputation for numerical variables. No duplicate rows were detected.
Main exclusion decision	All 13 baseline predictors were retained; one-year status, follow-up and target-derived fields were excluded.	`id` was excluded as an identifier-like column; sensitive attributes were retained for audit rather than used as predictors.
Strongest target-association screens	Association screens were used as review flags; no retained predictor was excluded automatically.	`age` (0.245), `heart_disease` (0.135), `avg_glucose_level` (0.132) and `hypertension` (0.128).
Proxy-review signal	Some retained baseline variables showed high association with audit variables, for example age with derived age group and CD8 or prior-treatment variables with sensitive labels. These signals were kept as audit flags rather than automatic exclusion rules.	Age was directly linked to the derived age group, and glucose/BMI showed proxy signals. These variables were clinically relevant, so the pipeline used them for prediction while requiring subgroup audit.
Decision supported	Retain baseline predictors for retrospective mortality analysis; audit proxy-sensitive information.	Retain clinically relevant risk factors, but do not interpret exclusion of sensitive attributes as sufficient to remove subgroup effects.

The feature screen therefore served two purposes. First, it prevented obvious leakage and non-predictive identifiers from entering the model. Second, it documented potential proxy behavior. Proxy scores did not automatically remove a clinically relevant variable, because removing age or disease history

can make a clinical model unusable. Instead, the score indicated that subgroup audit remained necessary even when protected attributes were not included in the predictor set.

4.3. Benchmark methods and experimental setup

The benchmark stage used conventional tabular ML families. In the ACTG175 mortality execution, the enabled models were logistic regression, elastic-net logistic regression, random forest, Extra Trees and XGBoost; the stroke-risk execution retained its previously reported tabular baseline registry. These models were selected because the case studies are structured tabular rare-event datasets, where linear and tree-based models offer strong baselines, reasonable sample efficiency and interpretable operating-point behaviour. Deep learning and transformer-based models were not used in the reported experiments because the datasets did not contain imaging, natural language or longitudinal sequences, and the low number of positive events would make high-capacity models difficult to justify without external validation. All model comparisons used the same train-validation-test split, preprocessing policy and validation-threshold sweep within each execution.

4.4. Evaluation metrics and uncertainty reporting

The evaluation reported both ranking metrics and thresholded operating metrics. ROC-AUC and average precision summarised ranking behaviour; accuracy, balanced accuracy, recall, precision, F1-score, false-positive rate, false-negative rate and selection rate described the selected threshold; Brier score and expected calibration error summarised probability calibration. For the selected baselines, 95% confidence intervals for thresholded metrics were estimated by bootstrap resampling of held-out test decisions. This quantification of uncertainty is most important for recall and balanced accuracy because the positive test sets contained only 25 events in ACTG175 and 50 events in the stroke-risk dataset.

4.5. Baseline modeling and threshold decisions

The split structure preserved event prevalence across training, validation and test partitions: ACTG175 had 25 events in 412 test records, and the stroke-risk dataset had 50 events in 1,022 test records. Hyperparameters were selected by stratified cross-validation using ROC-AUC. The probability threshold was then selected on the validation set by maximising balanced accuracy, rather than accepting a default 0.5 threshold. This choice was

important because both outcomes were rare and clinically relevant events could otherwise be missed.

As part of the experiments, the selected baseline in the ACTG175 mortality execution was logistic regression with isotonic probability calibration and a threshold of 0.07. In the stroke-risk dataset, XGBoost was selected [56] with a threshold of 0.06. Both thresholds were well below 0.5, reflecting the rare-event setting and the emphasis on sensitivity. These results are summarized in Table 5 and Figure 4.

Table 5: Selected baseline performance in the final execution. Values in parentheses are 95% confidence intervals where available from the exported execution output; ACTG175 average precision is reported from the exported `test_average_precision` field. BA = balanced accuracy; AP = average precision; ECE = expected calibration error; FPR = false-positive rate; FNR = false-negative rate.

**Test-set metric**	**ACTG175**	**Stroke-risk**
Selected model	Logistic regression	XGBoost
Test records / events	412 / 25	1,022 / 50
Threshold	0.07	0.06
Accuracy	0.646	0.731 (0.705–0.758)
BA	0.624 (0.552–0.651)	0.773 (0.714–0.826)
ROC-AUC	0.710 (0.608–0.787)	0.849
AP	0.320	0.282
Brier score	0.054 (0.044–0.056)	0.041
ECE	0.014	0.009
Recall / sensitivity	0.600	0.820 (0.706–0.922)
Precision	0.099	0.134 (0.096–0.173)
F1-score	0.170	0.230 (0.170–0.289)
FPR	0.351 (0.305–0.401)	0.274 (0.245–0.301)
FNR	0.400 (0.306–0.538)	0.180 (0.078–0.294)
Selection rate	0.367	0.300 (0.272–0.329)

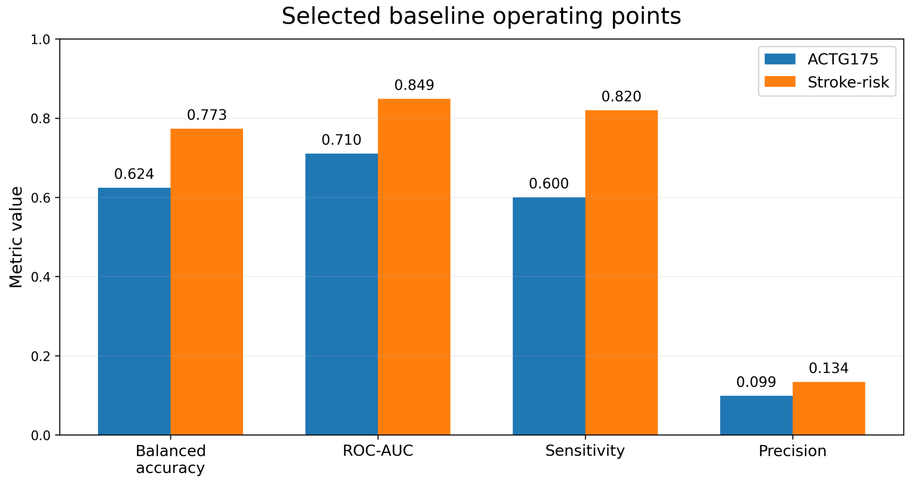

Figure 4: Selected baseline operating points in the two case studies. Balanced accuracy, ROC-AUC, sensitivity and precision are shown because clinical interpretation requires both ranking quality and thresholded behaviour.

The operating-point decision illustrates why the pipeline separates ranking quality from clinical classification. ROC-AUC described how well the model ranked patients, but the selected threshold determined who would be flagged. The selected thresholds flagged 36.7% of ACTG175 test records and 30.0% of stroke-risk test records. These selection rates imply a substantial follow-up burden, and the low precision values mean that most flagged patients did not experience the observed event. Therefore, the baselines were useful for retrospective benchmarking and fairness stress-testing, not sufficient for deployment approval.

4.6. Fairness audit decisions

The subgroup audit showed that global discrimination did not imply uniform error behaviour. For the selected ACTG175 mortality baseline, as seen in Figure 5, the largest single-attribute false-negative-rate gap was observed for gender label (0.417), which was therefore selected as the primary fairness priority in the ACTG175 execution. The combined FPR+FNR gaps were 0.457 for gender label, 0.260 for age group, 0.165 for homosexual-activity label, 0.145 for drug-use label and 0.129 for race label. The component view showed that the gender gap was mainly driven by missed mortality events: female records had a false-negative rate of 0.750 compared with 0.333 for male records. In the stroke-risk execution, as seen in Figure 6, age group and

work type dominated the single-attribute fairness profile, with combined gaps of 1.522 and 1.521, respectively. The component view showed that these large gaps were dominated by false-negative differences in addition to false-positive differences, reflecting the difficulty of detecting rare positive cases in younger and lower-risk subgroups. Age-related gaps were expected to be large because stroke prevalence is strongly age-dependent, but the pipeline still flagged the behaviour as clinically relevant because younger positive cases could be under-detected.

Intersectional audits produced the largest numerical gaps in both datasets. This was expected because intersectional groups divide the cohort into smaller and more specific patient profiles. The combined gap used by the pipeline ranges from 0 to 2 because both the false-positive-rate gap and the false-negative-rate gap can range from 0 to 1. Therefore, a value of 2.000 represents the maximum possible observed separation in thresholded errors. However, the worst ACTG175 intersection had a minimum subgroup size of 1, and the worst stroke-risk intersection had a minimum subgroup size of 2. With such small denominators, a single correct or incorrect classification can move a subgroup rate from 0 to 1. These values were therefore not interpreted as stable estimates of unfairness. They were interpreted as support warnings: the available data were too sparse to make robust intersectional fairness claims, and additional data collection, cohort harmonization or prospective monitoring would be required before such claims could be defended clinically.

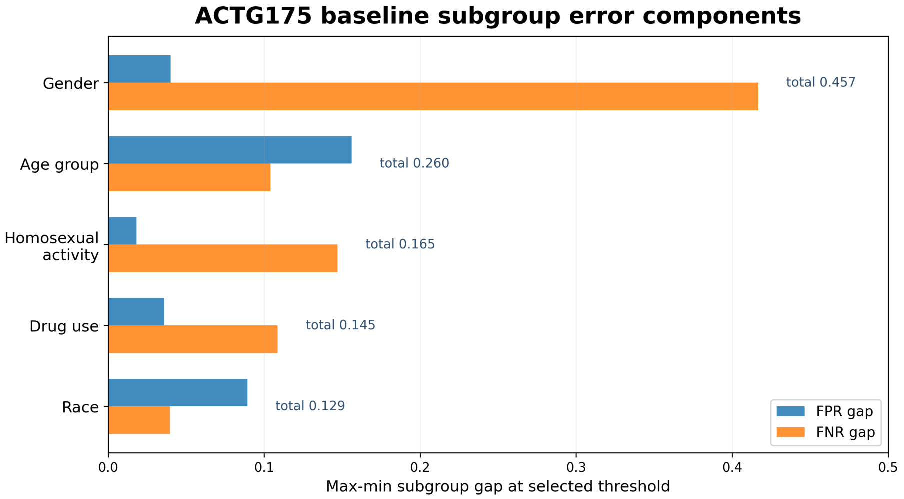

Figure 5: ACTG175 single-attribute subgroup error components at the selected baseline. Paired bars show the false-positive-rate gap and false-negative-rate gap separately across groups within the same audited attribute after correction to the death-only endpoint; labels report the combined total.

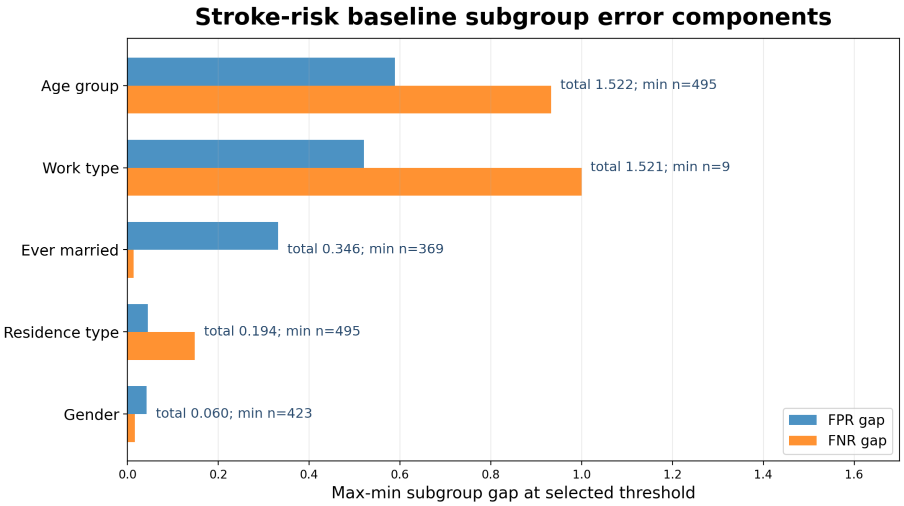

Figure 6: Stroke-risk single-attribute subgroup error components at the selected baseline. Paired bars show the false-positive-rate gap and false-negative-rate gap separately across groups within the same audited attribute; labels report the combined total and the minimum subgroup size.

The fairness decision was therefore a two-level one. Single-attribute

gaps were considered more interpretable when group sizes were adequate. Intersectional gaps were preserved because they can reveal hidden risks, but very small subgroups were labelled as unstable. This distinction is clinically important: the pipeline does not allow a large numerical gap based on one or two patients to be treated as a definitive subgroup conclusion. The selection-rate audit added a resource-allocation perspective. In the ACTG175 dataset, the primary gender-label comparison selected 31.4% of female records and 37.7% of male records for follow-up at the selected threshold. In the stroke-risk dataset, selection rates ranged from 0.0% in children or never-worked subgroups to 62.0% in participants aged 45 years or older. These values were not interpreted as direct clinical policy recommendations, but they show how thresholded prediction can concentrate follow-up burden in particular groups.

4.7. Mitigation evidence and final recommendations

The mitigation stage compared pre-processing, in-processing, and post-processing options. The two datasets favored different mitigation families as seen in Table 6. In the ACTG175, the strongest 3×3 held-out comparison for the primary fairness target was elastic-net logistic regression combined with Fairlearn demographic-parity threshold optimisation. The stroke-risk dataset favored threshold post-processing applied to a random forest. This difference supports the pipeline design: mitigation is handled as a comparative laboratory rather than a fixed recipe.

Table 6: Leading mitigation candidates and recommendation evidence. BA = balanced accuracy; LR = logistic regression; RF = random forest; DP = demographic parity. Bootstrap support refers to the aggregate combined FPR+FNR gap reduction.

Dataset	Leading mitigation candidate	Evidence signal	Recommendation
ACTG175	Elastic-net LR + Fairlearn DP threshold optimisation.	Primary gender-label combined FPR+FNR gap improved by 0.190 in the 3×3 held-out comparison, corresponding to an approximate 55.8% relative reduction, with BA change +0.046 versus the logistic benchmark.	Use as benchmark and further validation evidence. The execution does not by itself establish deployment readiness; calibration review, stronger bootstrap evidence and external validation remain required.
Stroke-risk	RF with Fairlearn DP threshold post-processing.	Aggregate combined FPR+FNR gap reduction of 39.41%; mean BA change -0.002; aggregate bootstrap p = 0.00399, so the aggregate reduction was statistically supported.	Validate the mitigation candidate externally. Clinical threshold review, calibration assessment and monitoring planning remain required.

The supplementary material reproduces the full model-by-mitigation ranking from artefact 20 for both datasets and includes the selected baseline calibration values and subgroup error-component tables.

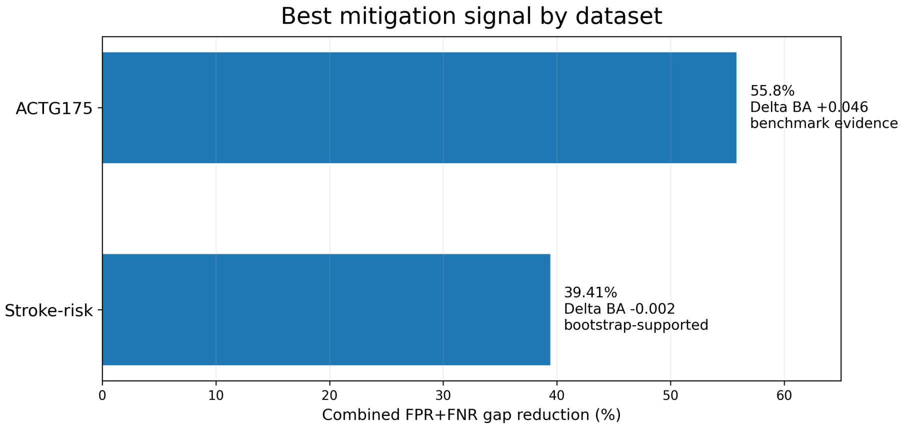

Figure 7: Best mitigation signal by dataset. The bars show the strongest reported mitigation signal for each dataset. ACTG175 reports the primary gender-label gap reduction from the death-only ACTG175 execution; stroke-risk reports the aggregate combined FPR+FNR gap reduction from the exported stroke execution. Labels report balanced-accuracy change.

The statistical-evidence table was used to separate numerical improvement from supported improvement. The ACTG175 showed a favourable numerical signal for the primary gender-label mitigation comparison, but this was interpreted conservatively because the execution output is a retrospective demonstration and does not by itself provide prospective or external validation. The recommendation is therefore conservative: the candidate is a useful benchmark for further validation, not a clinical solution. In the stroke-risk dataset, the leading candidate passed the performance guard and had bootstrap-supported aggregate gap reduction. Even in that case, the output is not deployment approval; it is a recommendation to validate the mitigation candidate externally and to review calibration, threshold consequences and monitoring plans.

The decision registry translated these outputs into reproducible recommendations. ACTG175 selected the fixed-horizon one-year mortality endpoint, 13 retained baseline predictors, logistic regression as baseline, isotonic calibration, a 0.07 threshold and gender label as the primary fairness priority. For the stroke-risk dataset, the registry selected the generic binary target `stroke`, six predictors, XGBoost as baseline and the marital-status-by-residence-by-work-type-by-age-group intersection as the primary stress point. In both cases, the pipeline classified the evidence as retrospective audit evidence requiring further validation rather than as direct clinical deployment evidence.

5. Discussion and limitations

The main contribution of FADOP is to make fairness-aware data orchestration understandable and reproducible for biomedical binary prediction. The pipeline does not replace clinical judgement. Instead, it organises the evidence that clinicians, data scientists and governance teams need before a model can be trusted.

This contribution is aligned with the needs of smart health. Smart-health systems often depend on heterogeneous data sources, automated workflows and rapid model updating. In that context, a model-performance table is not enough. The data pathway must be documented, the target must be clinically justified, errors must be interpreted by subgroup and mitigation must be evaluated as a trade-off rather than assumed beneficial. FADOP responds to this need by producing a structured evidence package rather than a single best model.

The case studies show why fairness should be evaluated at the level of clinical decisions. ROC-AUC describes how well a model ranks patients by risk, but it does not show what happens after a threshold is applied. Patients are affected by thresholded decisions: alerts, referrals, closer monitoring or reassurance. Therefore, sensitivity, false positives and false negatives must be reviewed at the operating point. This is especially important for rare outcomes, because the positive class is small and clinically consequential.

Another important finding is that protected-attribute removal is not a sufficient fairness strategy. Even when sensitive variables are excluded from the predictor set, subgroup information may remain encoded through other variables. The appropriate approach is therefore to avoid unnecessary use of sensitive variables for prediction while still using them for audit, interpretation and governance [75–79]. This distinction is essential in medical datasets, where fairness auditing cannot be performed if relevant subgroup information is absent or undocumented.

The recommendation layer is deliberately conservative. A mitigation candidate may improve the measured fairness gap, but deployment also requires external validation, calibration assessment, clinical threshold justification, privacy review, monitoring and human oversight. This position is consistent with health-AI governance recommendations from WHO and medical-device good ML practice initiatives [80–82]. The EU AI Act similarly emphasises risk management, data quality, technical documentation and human oversight for high-risk AI systems [83].

5.1. Limitations

The current version of FADOP focuses on binary classification and fixed-horizon transformation of survival-style data. This scope is deliberate because many clinical risk tasks can be expressed as event versus no-event, but it remains restrictive. Future extensions should address time-to-event modelling, multi-class prediction, longitudinal data, imaging-specific quality control, wearable-signal artifact removal, omics harmonisation and federated privacy-preserving settings.

The case studies used in our experiments were retrospective and based on publicly available datasets. They show workflow operation, not clinical efficacy. Additional validation would be required before any model derived through the pipeline could be considered for use in practice. Subgroup sparsity is also a central limitation. The pipeline can detect when intersectional groups are too small to support stable conclusions, but it cannot solve insufficient representation by computation alone.

A related limitation of the current AI Readiness Assessment is that it does not explicitly perform upfront sample-size adequacy testing or dynamic statistical power calculations prior to model training. While FADOP actively addresses small-sample vulnerabilities during downstream evaluation and gap analysis by reporting subgroup support counts and raising automated low-sample warnings, formal pre-training sample-size sizing remains outside the present pipeline. To guide users in evaluating initial dataset suitability, practical guidelines based on model complexity should be considered: in classical clinical epidemiology, linear baselines traditionally aim for an Events Per Variable ratio (EPV $= E/p$, where E represents the minority event count and p denotes feature count) of at least 10–20 to prevent parameter instability [84, 85]. However, high-dimensional or rare-event biomedical tasks frequently operate below these traditional EPV thresholds. To handle varying sample-size regimes safely, FADOP employs model-appropriate safeguards: L1/L2-regularized classifiers are integrated to constrain parameter variance in low-EPV settings, whereas non-linear tree ensembles, which require higher absolute event counts ($E \geq$ 100–200) to fit interaction terms without severe overfitting—are reserved for higher-capacity datasets. Incorporating automated EPV checks and empirical sample-size adequacy heuristics directly into the initial readiness phase represents an important direction for future work.

The scope also excludes formal explainability analysis. Although the pipeline reports feature policy, proxy screening and model-family rationale, it

does not yet provide SHAP values, counterfactual explanations, rule extraction or clinician-facing explanation reports. Explainability is a necessary future component because clinical AI users need to understand why a model flags a patient, not only whether the model is accurate or fairness-audited. Similarly, deep learning and transformer models were outside the present tabular rare-event demonstration. They should be considered in future versions when the input modality justifies them, for example imaging, text or longitudinal physiological streams, and when sample size and external validation support their use. Finally, ACTG175 retained randomized treatment arm for a retrospective post-randomisation mortality demonstration; a treatment-excluded sensitivity analysis should be added before any prospective pre-treatment use.

Further work should focus on clinical usability. This might include richer visual dashboards, patient-safety oriented threshold selection, formal calibration review, decision-curve analysis, model-interpretability reports and prospective evaluation with clinicians. These extensions would strengthen the link between fairness-aware modelling and real clinical decision support.

6. Conclusions and future work

The proposed FADOP provides a clinically oriented data-orchestration pipeline for biomedical binary classification. It systematically evaluates AI-readiness, endpoint validity, baseline prediction, subgroup error patterns, mitigation options and final recommendations. The two case studies reported in our experiments demonstrate that the same workflow can be applied to different biomedical datasets, including a corrected ACTG175 one-year mortality endpoint, and can generate comparable evidence without overclaiming deployment readiness. By linking each step to the method applied, the output generated and the decision supported, the pipeline is positioned as a practical contribution for smart-health data orchestration, reproducible biomedical AI and fairness-aware clinical prediction. Future work should extend the pipeline to time-to-event models, multimodal data, model explainability, automated pre-training sample-size and EPV adequacy checks, prospective clinician-in-the-loop evaluation and stronger dataset-provenance capture for public datasets without immutable versioning.

Ethics statement

Ethics and Consent to Participate declarations: not applicable. No new human-subject data were collected. The case-study executions used public

datasets and were conducted for methodological demonstration.

Data availability

ACTG175 is available through the UCI Machine Learning Repository [73]. The stroke-risk dataset is available through Kaggle at `https://www.kaggle.com/datasets/ranaghulamnabi/stroke-risk-dataset` [74]. Raw data redistribution should follow the conditions specified by the respective repositories. For Kaggle datasets without immutable version identifiers, users should retain the downloaded file, access date, licence note and file hash in their governed local execution package.

Code availability

The reusable source code and citable repository snapshot are available as: Marta Alberola Martin, *FADOP (Fairness-Aware Data Orchestration Pipeline)* (version "fair"), Zenodo, 2026, DOI: `https://doi.org/10.5281/zenodo.20368455` [86]. The archived material is released under the Creative Commons Attribution 4.0 International licence (CC BY 4.0). The repository contains the modular `fairness_pipeline/` source package, execution scripts, requirements files, figures, final-execution tables, support audits and reproducibility artifacts. Raw datasets are not redistributed in the code package; the workflow expects locally governed dataset access and exports aggregate audit outputs, decision registries and reproducibility tables.

Funding

This study was funded by Spanish PID2022-143299OB-I00/AEI/10.13039/501100011033/FEDER, UE and by Fundació La Marató de TV3, La Marató 2015, Diabetis i Obesitat (grant 201633.10), and projects PI18/00518 and PI21/01384, funded by ISCIII and co-funded by the European Union.

Declaration of competing interest

The authors declare that they have no potential conflicts of interest.

Author contributions

M.A. software design and development, M.A, C.K and P.C research, M.A. data analysis, M.A writing of the main manuscript. All authors reviewed the manuscript.

References

[1] M. D. Wilkinson, M. Dumontier, I. J. Aalbersberg, G. Appleton, M. Axton, A. Baak, N. Blomberg, J.-W. Boiten, L. Bonino da Silva Santos, P. E. Bourne, et al., The FAIR guiding principles for scientific data management and stewardship, Scientific Data 3 (2016) 160018. `doi:10.1038/sdata.2016.18`.

[2] T. Gebru, J. Morgenstern, B. Vecchione, J. W. Vaughan, H. Wallach, H. Daume III, K. Crawford, Datasheets for datasets, Communications of the ACM 64 (12) (2021) 86–92. `doi:10.1145/3458723`.

[3] M. Mitchell, S. Wu, A. Zaldivar, P. Barnes, L. Vasserman, B. Hutchinson, E. Spitzer, I. D. Raji, T. Gebru, Model cards for model reporting, in: Proceedings of the Conference on Fairness, Accountability, and Transparency, 2019, pp. 220–229. `doi:10.1145/3287560.3287596`.

[4] I. D. Raji, A. Smart, R. N. White, M. Mitchell, T. Gebru, B. Hutchinson, J. Smith-Loud, D. Theron, P. Barnes, Closing the AI accountability gap: Defining an end-to-end framework for internal algorithmic auditing, in: Proceedings of the 2020 Conference on Fairness, Accountability, and Transparency, 2020, pp. 33–44. `doi:10.1145/3351095.3372873`.

[5] Z. Obermeyer, B. Powers, C. Vogeli, S. Mullainathan, Dissecting racial bias in an algorithm used to manage the health of populations, Science 366 (6464) (2019) 447–453. `doi:10.1126/science.aax2342`.

[6] R. Correa, M. Shaan, H. Trivedi, B. Patel, L. A. G. Celi, J. W. Gichoya, I. Banerjee, A systematic review of "fair" AI model development for image classification and prediction, Journal of Medical and Biological Engineering 42 (2022) 816–827. `doi:10.1007/s40846-022-00754-z`.

[7] Y. Yang, M. Lin, H. Zhao, Y. Peng, F. Huang, Z. Lu, A survey of recent methods for addressing AI fairness and bias in biomedicine, Journal of

Biomedical Informatics 154 (2024) 104646. doi:10.1016/j.jbi.2024.104646.

[8] M. Liu, et al., A scoping review and evidence gap analysis of clinical AI fairness, npj Digital Medicine (2025). doi:10.1038/s41746-025-01667-2.

[9] E. J. Topol, High-performance medicine: the convergence of human and artificial intelligence, Nature medicine 25 (1) (2019) 44–56.

[10] M. Komorowski, L. A. Celi, O. Badawi, A. C. Gordon, A. A. Faisal, The artificial intelligence clinician learns optimal treatment strategies for sepsis in intensive care, Nature medicine 24 (11) (2018) 1716–1720.

[11] C. König, P. Copado, A. Vellido, À. Nebot, C. Angulo, M. Lamarca, V. Acuña, F. Berna, S. Moritz, Ł. Gawęda, et al., An artificial intelligence-based platform for personalized predictions of metacognitive training effectiveness, Computational and structural biotechnology journal 28 (2025) 281–293.

[12] C. Dwork, M. Hardt, T. Pitassi, O. Reingold, R. Zemel, Fairness through awareness, in: Proceedings of the 3rd Innovations in Theoretical Computer Science Conference, 2012, pp. 214–226. doi:10.1145/2090236.2090255.

[13] M. Hardt, E. Price, N. Srebro, Equality of opportunity in supervised learning, in: Advances in Neural Information Processing Systems, Vol. 29, 2016.

[14] N. Mehrabi, F. Morstatter, N. Saxena, K. Lerman, A. Galstyan, A survey on bias and fairness in machine learning, ACM Computing Surveys 54 (6) (2021) 1–35. doi:10.1145/3457607.

[15] S. Barocas, A. D. Selbst, Big data's disparate impact, California Law Review 104 (2016) 671–732. doi:10.2139/SSRN.2477899.

[16] A. D. Selbst, D. Boyd, S. A. Friedler, S. Venkatasubramanian, J. Vertesi, Fairness and abstraction in sociotechnical systems, in: Proceedings of the Conference on Fairness, Accountability, and Transparency, 2019, pp. 59–68. doi:10.1145/3287560.3287598.

[17] C. König, M. I. Cardenas, P. J. Copado, A. Vellido, Fairness-aware machine learning for biomedical prediction: Evaluating and correcting bias in gallstone diagnosis models, in: Conference of the Spanish Society of Artificial Intelligence in Biomedicine, Springer, 2025, pp. 59–73.

[18] A. Chouldechova, Fair prediction with disparate impact: A study of bias in recidivism prediction instruments, Big Data 5 (2) (2017) 153–163. doi:10.1089/big.2016.0047.

[19] J. Kleinberg, S. Mullainathan, M. Raghavan, Inherent trade-offs in the fair determination of risk scores, in: 8th Innovations in Theoretical Computer Science Conference, 2017. doi:10.4230/LIPICS.ITCS.2017.43.

[20] S. Corbett-Davies, E. Pierson, A. Feller, S. Goel, A. Huq, Algorithmic decision making and the cost of fairness, in: Proceedings of the 23rd ACM SIGKDD International Conference on Knowledge Discovery and Data Mining, 2017, pp. 797–806. doi:10.1145/3097983.3098095.

[21] Y. Zong, Y. Yang, T. Hospedales, MEDFAIR: Benchmarking fairness for medical imaging, in: International Conference on Learning Representations, 2023.

[22] C. Jones, M. Roschewitz, B. Glocker, The role of subgroup separability in group-fair medical image classification, in: MICCAI Workshop on Fairness of AI in Medical Imaging, 2023, arXiv:2307.02791.

[23] E. Puyol-Anton, B. Ruijsink, S. K. Piechnik, S. Neubauer, S. E. Petersen, R. Razavi, A. P. King, Fairness in cardiac MR image analysis: An investigation of bias due to data imbalance in deep learning based segmentation, in: International Workshop on Statistical and Neural Learning for Biomedical Image Analysis, 2021, arXiv:2106.12387.

[24] B. Li, X. Shi, H. Gao, X. Jiang, K. Zhang, A. O. Harmanci, et al., Enhancing fairness in disease prediction by optimizing multiple domain adversarial networks, bioRxiv (2023). doi:10.1101/2023.08.04.551906.

[25] T. D. Hoang, C.-H. Hsu, J. Park, A. Singh, X. Zhang, X. Lin, et al., Enabling end-to-end secure federated learning in biomedical research on heterogeneous computing environments with APPFLx, Computational

and Structural Biotechnology Journal 28 (2025) 29–39. doi:10.1016/j.csbj.2024.12.001.

[26] A. Tajabadi, A. Idris, E. A. AlOmar, R. M. Alzahrani, M. M. AlGhamdi, M. S. Hossain, G. Muhammad, Privacy-preserving decentralized learning methods for biomedical applications, Computational and Structural Biotechnology Journal 23 (2024) 3281–3287. doi:10.1016/j.csbj.2024.08.024.

[27] S. Madathil, D. Jayathilake, T. Ekanayake, S. Kafle, S. Shahi, S. Karunasekara, et al., Revolutionizing healthcare data analytics with federated learning: A comprehensive survey of applications, systems, and future directions, Computational and Structural Biotechnology Journal 28 (2025) 217–238. doi:10.1016/j.csbj.2025.06.009.

[28] C. König, P. Jesús Copado, C. Angulo, À. Nebot, A. Vellido, Evaluating privacy preservation in regression models for a small medical dataset, in: International Work-Conference on the Interplay Between Natural and Artificial Computation, Springer, 2026, pp. 381–390.

[29] D. H. Kim, S. Y. Lee, S. Lee, S. Kim, T. Shon, D. Shin, T. M. Park, Self-sovereign management scheme of personal health record with personal data store and decentralized identifier, Computational and Structural Biotechnology Journal 28 (2025) 16–28. doi:10.1016/j.csbj.2024.11.036.

[30] F. Folkvord, N. Rudiger, P. Decorte, M. Boysen, J. Ainsworth, S. Casteleyn, et al., Using patient-generated health data more efficiently/effectively to facilitate the implementation of value-based healthcare in the EU: Innovation report, Computational and Structural Biotechnology Journal 24 (2024) 672–678. doi:10.1016/j.csbj.2024.10.026.

[31] V. C. Pezoulas, K. Kourou, F. C. Kalatzis, T. P. Exarchos, E. Kourou, D. I. Fotiadis, Synthetic data generation methods in healthcare: A review on open-source tools and methods, Computational and Structural Biotechnology Journal 23 (2024) 2892–2910. doi:10.1016/j.csbj.2024.07.005.

[32] V. Nisevic, R. S. S. Guizzardi, M. Tomic, S. Mitrovic, M. Zdravkovic, et al., Synthetic data in medicine: Legal and ethical considerations for

patient profiling, Computational and Structural Biotechnology Journal 28 (2025) 190–198. doi:10.1016/j.csbj.2025.05.026.

[33] J. Cheng, Y. Ma, X. Zhang, X. Xu, S. Wang, D. Zhang, et al., Instance-level medical image classification for text-based retrieval in a medical data integration center, Computational and Structural Biotechnology Journal 24 (2024) 434–450. doi:10.1016/j.csbj.2024.06.006.

[34] Z. Wu, V. H. Koelzer, Towards generative digital twins in biomedical research, Computational and Structural Biotechnology Journal 24 (2024) 565–573. doi:10.1016/j.csbj.2024.09.030.

[35] Y. Huang, M. M. Hossain, X. Huang, R. Zhang, From explainable to interpretable deep learning for natural language processing in healthcare: How far from reality?, Computational and Structural Biotechnology Journal 24 (2024) 362–373. doi:10.1016/j.csbj.2024.05.004.

[36] S. U. Rehman, K. N. Qureshi, M. H. Alsharif, A. Nadeem, K. M. Malik, et al., IBS-ECDHE: A blockchain-enhanced lightweight protocol for secure cloud-IoT in biomedical healthcare cyber-physical systems, Computational and Structural Biotechnology Journal (2025). doi:10.1016/j.csbj.2025.10.059.

[37] R. K. E. Bellamy, K. Dey, M. Hind, S. C. Hoffman, S. Houde, K. Kannan, P. Lohia, J. Martino, S. Mehta, A. Mojsilovic, et al., AI Fairness 360: An extensible toolkit for detecting and mitigating algorithmic bias, IBM Journal of Research and Development 63 (4/5) (2019) 4:1–4:15. doi:10.1147/JRD.2019.2942287.

[38] P. Saleiro, B. Kuester, L. Hinkson, J. London, A. Stevens, A. Anisfeld, K. T. Rodolfa, Aequitas: A bias and fairness audit toolkit, arXiv:1811.05577 (2018).

[39] S. Bird, M. Dudik, R. Edgar, B. Horn, R. Lutz, V. Milan, M. Sameki, H. Wallach, K. Walker, Fairlearn: A toolkit for assessing and improving fairness in AI, microsoft Technical Report MSR-TR-2020-32 (2020).

[40] H. Weerts, M. Dudik, R. Edgar, A. Jalali, R. Lutz, M. Madaio, H. Wallach, Fairlearn: Assessing and improving fairness of ai systems (2023). doi:10.48550/ARXIV.2303.16626.

[41] T. Hastie, R. Tibshirani, J. Friedman, The Elements of Statistical Learning: Data Mining, Inference, and Prediction, 2nd Edition, Springer, 2009.

[42] G. James, D. Witten, T. Hastie, R. Tibshirani, An Introduction to Statistical Learning: With Applications in R, 2nd Edition, Springer, 2021.

[43] R. Kohavi, A study of cross-validation and bootstrap for accuracy estimation and model selection, in: Proceedings of the Fourteenth International Joint Conference on Artificial Intelligence, 1995, pp. 1137–1145.

[44] B. Efron, R. J. Tibshirani, An Introduction to the Bootstrap, Chapman and Hall/CRC, 1994.

[45] Q. McNemar, Note on the sampling error of the difference between correlated proportions or percentages, Psychometrika 12 (2) (1947) 153–157. doi:10.1007/BF02295996.

[46] K. Pearson, Note on regression and inheritance in the case of two parents, Proceedings of the Royal Society of London 58 (1895) 240–242.

[47] H. Cramer, Mathematical Methods of Statistics, Princeton University Press, 1946.

[48] A. Agresti, An Introduction to Categorical Data Analysis, 3rd Edition, Wiley, 2018.

[49] F. Pedregosa, G. Varoquaux, A. Gramfort, V. Michel, B. Thirion, O. Grisel, M. Blondel, P. Prettenhofer, R. Weiss, V. Dubourg, J. Vanderplas, A. Passos, D. Cournapeau, M. Brucher, M. Perrot, E. Duchesnay, Scikit-learn: Machine learning in Python, Journal of Machine Learning Research 12 (2011) 2825–2830.

[50] D. W. Hosmer, S. Lemeshow, R. X. Sturdivant, Applied Logistic Regression, 3rd Edition, Wiley, 2013.

[51] H. Zou, T. Hastie, Regularization and variable selection via the elastic net, Journal of the Royal Statistical Society: Series B (Statistical Methodology) 67 (2) (2005) 301–320. doi:10.1111/j.1467-9868.2005.00503.x.

[52] L. Breiman, Random forests, Machine Learning 45 (1) (2001) 5–32. doi:10.1023/A:1010933404324.

[53] P. Geurts, D. Ernst, L. Wehenkel, Extremely randomized trees, Machine Learning 63 (1) (2006) 3–42. doi:10.1007/s10994-006-6226-1.

[54] J. H. Friedman, Greedy function approximation: A gradient boosting machine, The Annals of Statistics 29 (5) (2001) 1189–1232. doi:10.1214/aos/1013203451.

[55] C. Cortes, V. Vapnik, Support-vector networks, Machine Learning 20 (3) (1995) 273–297. doi:10.1007/BF00994018.

[56] T. Chen, C. Guestrin, Xgboost: A scalable tree boosting system, in: Proceedings of the 22nd acm sigkdd international conference on knowledge discovery and data mining, 2016, pp. 785–794.

[57] J. Platt, et al., Probabilistic outputs for support vector machines and comparisons to regularized likelihood methods, Advances in large margin classifiers 10 (3) (1999) 61–74.

[58] T. Fawcett, An introduction to ROC analysis, Pattern Recognition Letters 27 (8) (2006) 861–874. doi:10.1016/j.patrec.2005.10.010.

[59] H. He, E. A. Garcia, Learning from imbalanced data, IEEE Transactions on Knowledge and Data Engineering 21 (9) (2009) 1263–1284. doi:10.1109/TKDE.2008.239.

[60] A. R. Cartus, E. A. Samuels, M. Cerdá, B. D. Marshall, Outcome class imbalance and rare events: An underappreciated complication for overdose risk prediction modeling, Addiction 118 (6) (2023) 1167–1176.

[61] L. Zhao, Y. Leng, Y. Hu, J. Xiao, Q. Li, C. Liu, Y. Mao, Understanding decision curve analysis in clinical prediction model research, Postgraduate medical journal 100 (1185) (2024) 512–515.

[62] K. H. Brodersen, C. S. Ong, K. E. Stephan, J. M. Buhmann, The balanced accuracy and its posterior distribution, in: 2010 20th International Conference on Pattern Recognition, 2010, pp. 3121–3124. doi:10.1109/ICPR.2010.764.

[63] T. Saito, M. Rehmsmeier, The precision-recall plot is more informative than the ROC plot when evaluating binary classifiers on imbalanced datasets, PLOS ONE 10 (3) (2015) e0118432. doi:10.1371/journal.pone.0118432.

[64] G. W. Brier, Verification of forecasts expressed in terms of probability, Monthly Weather Review 78 (1) (1950) 1–3. doi:10.1175/1520-0493(1950)078<0001:VOFEIT>2.0.CO;2.

[65] C. Guo, G. Pleiss, Y. Sun, K. Q. Weinberger, On calibration of modern neural networks, in: Proceedings of the 34th International Conference on Machine Learning, 2017, pp. 1321–1330.

[66] F. Kamiran, T. Calders, Data preprocessing techniques for classification without discrimination, Knowledge and Information Systems 33 (1) (2012) 1–33. doi:10.1007/s10115-011-0463-8.

[67] N. V. Chawla, K. W. Bowyer, L. O. Hall, W. P. Kegelmeyer, SMOTE: Synthetic minority over-sampling technique, Journal of Artificial Intelligence Research 16 (2002) 321–357. doi:10.1613/jair.953.

[68] G. Lemaitre, F. Nogueira, C. K. Aridas, Imbalanced-learn: A Python toolbox to tackle the curse of imbalanced datasets in machine learning, Journal of Machine Learning Research 18 (17) (2017) 1–5.

[69] G. E. A. P. A. Batista, R. C. Prati, M. C. Monard, A study of the behavior of several methods for balancing machine learning training data, ACM SIGKDD Explorations Newsletter 6 (1) (2004) 20–29. doi:10.1145/1007730.1007735.

[70] A. Agarwal, A. Beygelzimer, M. Dudik, J. Langford, H. Wallach, A reductions approach to fair classification, in: Proceedings of the 35th International Conference on Machine Learning, 2018.

[71] Y. Huang, J. Guo, W.-H. Chen, H.-Y. Lin, H. Tang, F. Wang, H. Xu, J. Bian, A scoping review of fair machine learning techniques when using real-world data, medRxiv (2024). doi:10.1101/2024.03.03.24303669.

[72] S. M. Hammer, D. A. Katzenstein, M. D. Hughes, H. Gundacker, R. T. Schooley, R. H. Haubrich, W. K. Henry, M. M. Lederman,

J. P. Phair, M. Niu, M. S. Hirsch, T. C. Merigan, A trial comparing nucleoside monotherapy with combination therapy in hiv-infected adults with CD4 cell counts from 200 to 500 per cubic millimeter, The New England Journal of Medicine 335 (15) (1996) 1081–1090. doi:10.1056/NEJM199610103351501.

[73] S. Hammer, D. Katzenstein, M. Hughes, H. Gundacker, R. Schooley, R. Haubrich, W. K. Henry, M. Lederman, J. Phair, M. Niu, M. Hirsch, T. Merigan, AIDS Clinical Trials Group Study 175, dataset (2023). doi:10.24432/C5ZG8F.

[74] Rana Ghulam Nabi, Stroke risk dataset, https://www.kaggle.com/datasets/ranaghulamnabi/stroke-risk-dataset, kaggle dataset upload ranaghulamnabi/stroke-risk-dataset; accessed 5 June 2026 for the final execution package. Immutable version identifier and formal license were not exposed in the local execution metadata; therefore, local file snapshot and hash retention are recommended before reuse. (2026).

[75] M. S. A. Lee, J. Singh, The landscape and gaps in open source fairness toolkits, in: Proceedings of the 2021 CHI Conference on Human Factors in Computing Systems, 2021. doi:10.1145/3411764.3445261.

[76] B. Richardson, J. Garcia-Gathright, S. F. Way, J. S. Thom, H. Cramer, Towards fairness in practice: A practitioner-oriented rubric for evaluating fair ml toolkits, in: Proceedings of the 2021 CHI Conference on Human Factors in Computing Systems, 2021. doi:10.1145/3411764.3445604.

[77] K. Holstein, J. W. Vaughan, H. Daume, M. Dudik, H. Wallach, Improving fairness in machine learning systems: What do industry practitioners need?, in: Proceedings of the 2019 CHI Conference on Human Factors in Computing Systems, 2019. doi:10.1145/3290605.3300830.

[78] M. Madaio, L. Stark, J. W. Vaughan, H. Wallach, Co-designing checklists to understand organizational challenges and opportunities around fairness in ai, in: Proceedings of the 2020 CHI Conference on Human Factors in Computing Systems, 2020. doi:10.1145/3313831.3376445.

[79] M. Madaio, L. Egede, H. Subramonyam, J. W. Vaughan, H. Wallach, Assessing the fairness of ai systems: Ai practitioners' processes, challenges, and needs for support, Proceedings of the ACM on Human-Computer Interaction (2022). doi:10.1145/3512899.

[80] World Health Organization, Ethics and governance of artificial intelligence for health: WHO guidance, World Health Organization, 2021.

[81] U.S. Food and Drug Administration, Health Canada, Medicines and Healthcare products Regulatory Agency, Good machine learning practice for medical device development: Guiding principles, joint guiding principles (2021).

[82] International Medical Device Regulators Forum, Good machine learning practice for AI-enabled medical devices, final document (2025).

[83] European Parliament and Council of the European Union, Regulation (EU) 2024/1689 of the european parliament and of the council laying down harmonised rules on artificial intelligence, artificial Intelligence Act (2024).

[84] P. Peduzzi, J. Concato, E. Kemper, T. R. Holford, A. R. Feinstein, A simulation study of the number of events per variable in logistic regression analysis, Journal of clinical epidemiology 49 (12) (1996) 1373–1379.

[85] R. D. Riley, K. I. Snell, J. Ensor, D. L. Burke, F. E. Harrell Jr, K. G. Moons, G. S. Collins, Minimum sample size for developing a multivariable prediction model: Part ii-binary and time-to-event outcomes, Statistics in medicine 38 (7) (2019) 1276–1296.

[86] M. Alberola Martin, FADOP (Fairness-Aware Data Orchestration Pipeline), citable repository snapshot (2026). doi:10.5281/zenodo.20368455.
